# Spatial Transcriptomics Decodes Breast Cancer Microenvironment Heterogeneity: From Multidimensional Dynamic Profiling to Precision Therapy Blueprint Construction

**DOI:** 10.3390/biom15081067

**Published:** 2025-07-24

**Authors:** Aolong Ma, Lingyan Xiang, Jingping Yuan, Qianwen Wang, Lina Zhao, Honglin Yan

**Affiliations:** Department of Pathology, Renmin Hospital of Wuhan University, Wuhan 430060, China

**Keywords:** spatial transcriptomics, breast cancer, heterogeneity, tumor microenvironment, progression and metastasis, therapy

## Abstract

Background: Breast cancer, the most prevalent malignancy among women worldwide, exhibits significant heterogeneity, particularly in the tumor microenvironment (TME), which poses challenges for treatment. Spatial transcriptomics (ST) has emerged as a transformative technology, enabling gene expression analysis while preserving tissue spatial architecture. This provides unprecedented insights into tumor heterogeneity, cellular interactions, and disease mechanisms, offering a powerful tool for advancing breast cancer research and therapy. This review aims to synthesize the applications of ST in breast cancer research, focusing on its role in decoding tumor heterogeneity, characterizing the TME, elucidating progression and metastasis dynamics, and predicting therapeutic responses. We also explore how ST can bridge molecular profiling with clinical translation to enhance precision therapy. The key scientific concepts of review included the following: We summarize the technological advancements in ST, including imaging-based and sequencing-based methods, and their applications in breast cancer. Key findings highlight how ST resolves spatial heterogeneity across molecular subtypes and histological variants. ST reveals the dynamic interplay between tumor cells, immune cells, and stromal components, uncovering mechanisms of immune evasion, metabolic reprogramming, and therapeutic resistance. Additionally, ST identifies spatial prognostic markers and predicts responses to chemotherapy, targeted therapy, and immunotherapy. We propose that ST serves as a hub for integrating multi-omics data, offering a roadmap for precision oncology and personalized treatment strategies in breast cancer.

## 1. Introduction

Globally, breast cancer represents the predominant form of malignancy affecting the female population, standing as the primary factor in oncology-related mortality statistics [1]. The choice of breast cancer treatment regimen depends mainly on its molecular subtype. Currently, the clinically common subtypes include luminal A type, luminal B type, human epidermal growth factor receptor-2 positive (HER2+), and triple-negative breast cancer (TNBC), and patients of different subtypes have different responses to treatment [2,3,4]. However, breast cancer heterogeneity is a key feature and is of great significance for tumorigenesis, diagnosis, and treatment modalities [5]. This heterogeneity significantly impacts clinical outcomes, leading to varied prognoses even among patients with identical molecular subtypes. This variability poses substantial challenges for treatment strategies. Extensive multi-omics research has demonstrated that intra- and inter-tumor heterogeneity are primary drivers of recurrence and drug resistance [6]. Therefore, identifying the specific heterogeneity of breast cancer is helpful for mining potential therapeutic targets and biomarkers and formulating more personalized treatment strategies for patients to improve the treatment effect [7].

Notably, the occurrence and development of breast cancer not only depend on the proliferation of tumor cells, but also on the significant heterogeneity in the surrounding tumor microenvironment (TME) [8]. The TME consists of numerous components, including tumor cells, immune cells, endothelial cells (EC), cancer-associated fibroblasts (CAF), adipocytes and extracellular matrix (ECM), etc. The interactions between these components and tumor cells are highly complex and diverse, jointly shaping the heterogeneity characteristics of breast cancer from multiple dimensions and having an extremely profound impact on the initiation, progression, and metastasis of cancer [9]. This heterogeneity manifests not only across different populations but also varies significantly over time and space [10]. Thus, in-depth exploration of the complexity and heterogeneity of the TME in breast cancer holds pivotal importance for dissecting the heterogeneity of breast cancer and formulating effective treatment strategies.

Traditionally, biological experimental techniques used for TME analysis, such as immunohistochemistry and multiplex immunofluorescence, can only target certain cell populations and are unable to conduct a comprehensive analysis of the highly heterogeneous TME. Immunohistochemistry, limited by the technology itself, can only analyze a limited number of markers each time, making it difficult to comprehensively and completely present the complex diversity and heterogeneity of the TME. Although multiplex immunofluorescence technology can visualize multiple biomarkers simultaneously, it cannot provide comprehensive and systematic genomic information. This makes it difficult for these technologies to fundamentally reveal the genetic basis and molecular mechanisms of cell–cell interactions in the TME [11]. With advances in sequencing technology, bulk RNA-seq now offers efficient, high-throughput analysis of gene expression in tissue samples, facilitating TME studies. However, the average gene expression levels obtained by this technology cannot capture the heterogeneity information of the TME [12]. The emergence of single-cell RNA sequencing (scRNA-seq) has made it possible to analyze gene expression at the single-cell level [13]. When analyzing the genomic, transcriptomic, and other information of individual cells, this technology can accurately analyze the molecular characteristics of cells, but it will lose the spatial location information of cells in the tissue, making it difficult to present the overall structure and function of the tissue [14]. In cancer research, understanding the spatial location relationship between tumor cells and surrounding immune cells and their interactions is crucial for the development of treatment strategies. In recent years, spatial transcriptomics (ST) has developed rapidly and, as a powerful tool, has brought unprecedented insights to the study of TME [15]. ST has significant advantages. It can accurately record the spatial locations of cells in tissue sections, through which the relative positions of tumor cells, immune cells, and stromal cells can be determined, enabling in-depth exploration of signal transduction and regional differences in the spatial dimension [16]. At the same time, by presenting the gene expression patterns of different regions of the tissue, it can reveal the functional zoning of the tissue and the organization of cell populations, clearly presenting the gene expression differences and cell distribution patterns of different tissues [17]. In addition, through in situ analysis, it can observe the gene expression associations of adjacent cells, infer cell–cell signal transduction and interactions, and assist in understanding the spatial relationship between tumor cells and immune cells, providing a basis for the selection of prognostic markers and the development of treatment strategies [18,19].

**Figure 1:**
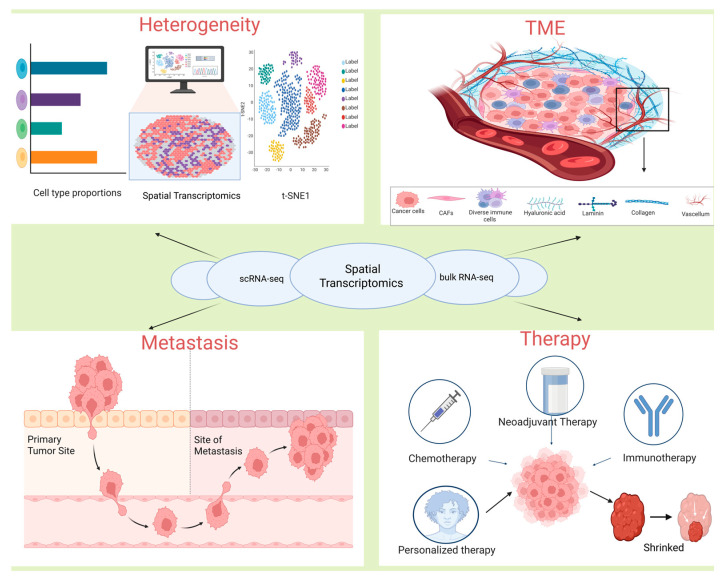
The applications of integrating ST with other technologies in tumor research.

As shown in Figure 1, this review systematically summarizes the core applications of ST in breast cancer, including the resolution of tumor molecular heterogeneity, construction of dynamic interaction networks within the TME, exploration of disease progression mechanisms, and prediction of therapeutic responses. By resolving tumor molecular heterogeneity, ST technology reveals regional gene expression differences and cellular subpopulation distribution patterns, while constructing complex spatial interaction networks among immune cells, stromal cells, and tumor cells. It further tracks dynamic changes in key signaling pathways during disease progression, thereby predicting patient responses to chemotherapy, targeted therapy, and immunotherapy. Through a multidimensional integration and in-depth analysis of ST and its groundbreaking progress in breast cancer research, the spatial characteristics of breast cancer heterogeneity have been emphasized. This has driven a transformation in treatment paradigms toward “spatial functional typing”, opening a new era for personalized diagnosis and therapy.

### 1.1. Overview of Spatial Transcriptomics Technology

**Figure 2:**
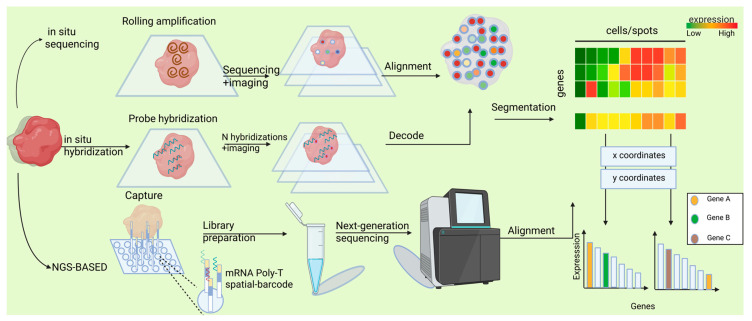
Spatial transcriptomics technologies generate a gene expression matrix. aIn situ sequencing methods directly decode transcript sequences within tissues. In situ hybridization techniques identify target sequences using complementary fluorescent probes.NGS-based spatial transcriptomic approaches assign location-specific barcodes to transcripts within a spot-based grid. The output of spatial transcriptomics is a gene expression matrix, where rows represent genes and columns denote spatial locations.

ST techniques can be broadly classified into two main categories: imaging-based and sequencing-based techniques [20,21]. The fundamental distinction between these two classes lies in their divergent methodologies for determining the spatial location and abundance of specific mRNA molecules within tissues. Imaging-based techniques leverage fluorescence characteristics and their corresponding intensities to decipher target genes and their respective abundances. Conversely, sequencing-based techniques rely on spatial barcodes present on arrays to retrieve the spatial positions of target genes in tissues and utilize next-generation sequencing (NGS) technologies to ascertain their expression levels (Figure 2). Under each of these two overarching techniques, numerous distinct sequencing methods can be further delineated.

#### 1.1.1. In Situ Approaches

Imaging-based ST employs in situ hybridization, where mRNA binds to labeled complementary probes, and transcripts are quantified through microscopy. For mRNA imaging, two primary methods distinguish between mRNA species [22]. The first is in situ sequencing (ISS), which directly sequences amplified mRNA within tissue samples using sequencing-by-ligation (SBL). The second uses fluorescently labeled gene-specific probes for hybridization, pairing single-stranded mRNA with complementary probes, a technique termed in situ hybridization (ISH). Thus, imaging-based methods primarily include ISH and ISS. ISS-based approaches directly decode transcript sequences within tissues by reverse transcribing RNA, performing rolling circle amplification, and conducting sequencing (Figure 2). This approach was pioneered by Ke et al. [23], who employed targeted probes for reverse transcription coupled with sequencing-by-ligation, enabling the study of approximately 50 targeted genes across diverse biological contexts including cancer [24], tuberculosis [25], and brain development [26]. Subsequent advancements led to the development of STARMap, which integrated innovations in hydrogel chemistry, optimized padlock and primer design, and introduced an error-resistant sequencing-by-ligation method, thereby facilitating the profiling of thousands of genes in the mouse cortex [27]. Parallel methodological developments, such as BaristaSeq [28] and Barseq [29] utilizing sequencing-by-synthesis, and HybISS [30] employing sequencing-by-hybridization, have achieved increased read lengths, enabling enhanced throughput and cellular barcoding capabilities. Notably, the integration of in situ sequencing with cDNA extraction for NGS [31] underscores the challenge in categorizing ST methods as exclusively NGS- or imaging-based. The potential for untargeted profiling through in situ sequencing has been demonstrated by FISSEQ [32], although this approach faces challenges of optical crowding and reduced sensitivity. Recent breakthroughs, exemplified by ExSeq, have shown that expansion microscopy can effectively address these limitations, enabling untargeted in situ sequencing in tissue samples [31].

The second category of imaging-based methods, ISH-based techniques, builds upon traditional ISH technologies by detecting target sequences through hybridization with complementary fluorescent probes (Figure 2). While initially constrained by the number of distinguishable transcripts, significant advancements have been achieved through the implementation of sequential hybridization and imaging rounds [33] combined with sophisticated barcoding strategies, enabling substantial multiplexing capabilities. MERFISH exemplifies this approach, utilizing successive hybridization rounds to detect fluorescently labeled probes, with serial images subsequently decoded using error-robust barcodes corresponding to specific transcript identities [34]. This technique has been successfully applied across various scales, from intracellular transcript [35] to tissue-level ST, such as in studies of the hypothalamic preoptic region [36]. An alternative strategy for increasing transcript detection capacity involves the use of pseudocolor combinations, as implemented in SeqFISH [37]. Similar to MERFISH, this method has proven valuable for investigating intracellular organization and generating comprehensive spatial maps, including those of the hippocampus [38]. Both methodologies have undergone significant refinement in recent years, achieving the capacity to detect approximately 10,000 genes at subcellular resolution [39]. Current research efforts continue to focus on enhancing the sensitivity and scalability of these techniques [40].

For both ISS- and ISH-based methodologies, image processing is essential for generating gene expression matrices. To obtain cell-level resolution, image segmentation is performed either manually for small areas or systematically using computational approaches. Watershed algorithms typically utilize DAPI-stained nuclei as reference points and define cell borders based on regions of low RNA density [41]. While these boundaries may not always correspond to actual physical cell borders, they effectively serve the purpose of assigning individual mRNA molecules to specific cells. Alternatively, data analysis can commence at the pixel level, incorporating gene expression information to delineate cellular boundaries [42,43,44].

#### 1.1.2. NGS-Based Approaches

The other major approach in ST involves extracting mRNAs from tissue while maintaining spatial information, followed by profiling mRNA species NGS techniques. This forms the basis of sequencing-based ST technologies, where “sequencing” refers to NGS rather than ISS. Spatial information is typically preserved through two primary methods: (1) direct capture and recording of location, such as via microdissection or microfluidics, and (2) ligation of mRNAs to spatially barcoded probes arranged in a microarray [22]. While both approaches enable spatially resolved transcriptomics, array-based methods offer superior scalability and compatibility with standard NGS workflows. Consequently, within sequencing-based technologies, we focus on array-based methods (Figure 2).

Sequencing-based ST technologies capture mRNAs from tissue, synthesize complementary DNAs (cDNAs), and quantify gene-specific sequences through NGS, while retaining positional information at the point of mRNA capture. An early precursor to these methods was LCM, which enabled transcriptomic profiling of specific tissue regions using microarrays [22]. This was followed in the 2000s by Tomo-seq, where cryosectioned tissues were subjected to RNA-seq profiling [45]. A similar approach, Geo-seq, applied scRNA-seq to tissue sections [46]. Modern microdissection techniques, such as Nanostring’s GeoMx DSP [47], achieved variable spatial resolution down to near-single-cell levels by using gene-specific photocleavable probes. When UV light is applied to a tissue region, the probes are released and sequenced. However, LCM-based methods face challenges such as laser-induced mRNA degradation and the need for multiple independent library preparations. To address these limitations, STRP-seq was developed, enabling 2D gene expression profiling across consecutive thin sections that are cross-sectioned at different angles and sequenced to reveal spatial gene expression patterns [48]. While these microdissection-based techniques provide valuable tools for unbiased, spatially resolved transcriptome profiling, they are constrained by limited spatial resolution, potential mRNA degradation during LCM, and the requirement for extensive sample processing [22].

In contrast, array-based methods for spatially barcoded mRNA capture emerged with the introduction of ST in 2016 [49]. In this approach, tissue is mounted over an array, allowing released mRNAs to be captured by spatially barcoded probes, converted to cDNA, and sequenced. Unlike ISH and some ISS methods, ST probes are not gene-specific but instead capture polyadenylated mRNAs in an untargeted manner. The spatial resolution of array-based methods is determined by the size of the capture area associated with each unique barcode, analogous to pixel size in imaging. ST initially featured 100 μm (center-to-center) capture areas [49], but its commercialization by 10× Genomics as Visium improved this to hexagonal 55 μm resolution [50]. Other advancements include Slide-seq, which employs arrays of 10 μm-diameter barcoded beads, with barcodes pre-determined before tissue mounting [51]. Slide-seqV2, an improved version with enhanced barcoding and enzymatic library preparation, achieves 30–50% of the transcriptomic recovery efficiency per bead compared to droplet-based single-cell transcriptomics (e.g., 10× Genomics), enabling detection of hundreds to thousands of genes per 10 μm pixel [52]. High-definition spatial transcriptomics (HDST) operates similarly to Slide-seqV2 but confines beads to etched wells on slides, achieving a spatial resolution of 2 μm [52]. Emerging technologies like Stereo-seq have pushed resolution even further by using barcoded rolling circle amplification (RCA) products deposited in wells spaced 0.5 μm apart [53]. Stereo-seq and other ultra-high-resolution techniques, such as PIXEL-seq [54], achieve mRNA recovery rates per unit area comparable to Visium [22]. Stereo-seq is currently being commercialized by BGI as part of its STOmics platform, which is in early access. Additionally, while most array-based methods are designed for fresh frozen tissues, some, like Visium FFPE, are compatible with formalin-fixed paraffin-embedded (FFPE) tissues, albeit with additional preparation steps and a gene-specific probe set (though still profiling the entire genome) [55].

An alternative to mounting tissue onto arrays is to “print” arrays onto tissue using microfluidic channels, as demonstrated by deterministic barcoding in tissue for spatial omics sequencing (DBiT-seq) [56]. In this method, barcodes are deposited along one axis of a tissue section and then perpendicularly, creating unique barcodes at each intersection point. The spatial resolution depends on the microfluidic channel diameter, which can range from 10 μm to 50 μm. This approach minimizes mRNA diffusion away from capture areas and allows simultaneous protein assessment by introducing oligonucleotide-tagged antibodies through microfluidic channels prior to processing.

A limitation of these methods is that capture areas do not align with the complex contours of cellular morphology, often resulting in mRNAs from a single cell being distributed across multiple pixels. Even when capture areas are smaller than a single cell (e.g., HDST), they still lack true single-cell resolution, as they only capture mRNA from a cell-sized area rather than individual cells. To address this, recent techniques like XYZeq [57] and sci-Space [58] use spatially barcoded arrays not for mRNA capture but for labeling intact cells, which are then dissociated and subjected to scRNA-seq with spatial barcodes. These methods benefit from established scRNA-seq technologies, with sci-Space detecting an average of ~1200 genes per cell. However, they operate at lower spatial resolutions, with sci-Space using 80 μm radius spots and XYZeq featuring 500 μm center-to-center distances.

Array-based methods offer distinct advantages and disadvantages when compared to ISH and ISS-based approaches. One significant advantage is their ability to profile larger tissue sections; for instance, Visium can analyze areas up to 6.5 mm × 6.5 mm, whereas seqFISH is limited to approximately 0.5 mm^2^. Additionally, by utilizing NGS rather than microscopy, array-based methods circumvent the need for complex image-processing pipelines. Furthermore, these methods are untargeted, enabling comprehensive profiling of the entire transcriptome in any organism that utilizes polyadenylated mRNA. However, they generally exhibit lower spatial resolution and mRNA recovery rates compared to ISH and ISS methods [22]. Another limitation is their reliance on a fixed array, which can result in transcripts from multiple cells being captured at the same spot. Consequently, sophisticated computational analyses are required to infer the specific cell types present at each location.

In summary, while laser capture microdissection and in situ imaging-based methods are costly and less efficient, they offer high spatial resolution. In contrast, NGS-based and computational reconstruction methods are more cost-effective and efficient for processing large-scale datasets, albeit with slightly lower spatial localization precision compared to the former.

Different types of ST techniques and their representative applications have been summarized in Table 1.

## 2. Application of ST in Breast Cancer

ST revolutionizes breast cancer research by preserving tissue architecture while mapping gene expression, enabling insights into tumor heterogeneity, microenvironment dynamics, and regional drug resistance mechanisms, advancing precision medicine. This integration of spatial and molecular data represents a major leap in understanding breast cancer biology and optimizing treatments [59].

### 2.1. Decoding Tumor Heterogeneity in Breast Cancer

Ductal carcinoma and lobular carcinoma are two prevalent forms of breast cancer. Given their distinct anatomical origins, epithelial cells in ductal and lobular regions are hypothesized to exhibit inherent gene expression disparities. Bhat-Nakshatri et al. [60] utilized ST to determine gene expression differences between ductal and lobular epithelial cells from the same donor at time points ten years apart. The study revealed the distribution patterns of specific epithelial subtypes and differentially expressed genes in ductal and lobular regions. Notably, MGP, ANXA1, TACSTD2, KRT14, KRT17, WFDC2, STAC2, and ALDH1A3 were upregulated in ductal epithelial cells, while APOD and SNORC were elevated in lobular epithelial cells. Multi-omics analysis of these 10 genes’ expression patterns, combined with pathway enrichment analysis, revealed significant enrichment of metabolic pathways in ductal epithelial cells. In contrast, lobular epithelial cells showed enrichment in the mitogen-activated protein kinase kinase (MAPKK) signaling pathway and the paired amphipathic helix protein Sin3a–histone deacetylase complex pathway. The study further identified PTBP1 (a regulator of mRNA processing and alternative splicing [61]) as exhibiting significant age-dependent changes in both ductal and lobular epithelial cells. Furthermore, PTBP1 was found to be overexpressed in all breast cancer subtypes. Further analysis revealed that 168 genes were downregulated in epithelial cells with age. This finding highlights the profound impact of aging on gene expression and signaling pathways in mammary epithelial cells, providing critical insights into the age-related mechanisms of breast cancer.

Mixed invasive ductal and lobular carcinoma (MDLC) is a specific subtype of breast cancer that combines features of both invasive ductal carcinoma and invasive lobular carcinoma. Shah et al. [62] employed ST, genomics, and single-cell profiling to investigate the molecular heterogeneity of MDLC, revealing significant differences in biological features, signaling pathways, and mutational profiles between ductal and lobular tumor regions. The study found that different regions of MDLC may have independent origins and unique clinically actionable mutations. The ductal regions exhibited activation of MYC signaling pathways and basal-like immunometabolic signatures, while lobular regions were enriched with ER signaling pathways and luminal-A features. These biological divergences directly correlated with clinical outcomes, as evidenced by ER-negative/triple-negative ductal regions being associated with worse metastatic progression. The study demonstrated that distinct tumor regions within MDLC may have independent origins, exemplified by APOBEC mutations in ductal regions versus HRD deficiency in lobular regions of MDLC-3. Spatial specificity was also observed in CDH1 inactivation mechanisms, involving either genetic mutations or epigenetic regulation across different cases. Clinically, this spatial heterogeneity drove differential therapeutic responses: NOTCH pathway mutations in ductal regions contributed to endocrine therapy resistance, while ER signaling aberrations in lobular regions potentially compromised treatment efficacy. Notably, DNA repair gene mutations spanning both regions suggested PARP inhibitor sensitivity. These findings highlighted the importance of integrating spatial molecular profiling in MDLC management, proposing targeted strategies like ROS1 inhibitors for CDH1-deficient regions and providing a novel rationale for personalized clinical decision-making. Collectively, these studies elucidate ductal-lobular heterogeneity in both normal mammary glands and breast cancer and expound on the spatial heterogeneity of the ductal and lobular tissue regions, contributing to a profound understanding of the molecular evolution process of mammary tissues from a normal state to cancerous transformation.

Beyond developmental and neoplastic contexts, ST applications extend to molecular subtyping. Yoshitake et al. [63] utilized ST and scRNA-seq to uncover the spatial distribution of distinct functional compartments within ER+ breast cancer and their roles in tumor growth. The study revealed that proliferative cell populations, predominantly localized in the tumor core, served as the key drivers of luminal B breast cancer. In contrast, estrogen-responsive cell populations, primarily distributed in the peripheral regions, were not directly associated with tumor progression. Furthermore, the spatial characteristics of hypoxia-induced and inflammation-related compartments highlighted the complexity of the tumor microenvironment, providing a novel molecular foundation for the precision treatment of luminal breast cancer.

Chu et al. [64] pioneered the use of ST to uncover significant differences in gene expression profiles between younger and older patients with hormone receptor-positive (HR-positive) breast cancer, highlighting the critical role of age in breast cancer biology and treatment. The study revealed that PRSS23, SERPINA1, and ribosome-related genes are significantly upregulated in younger patients, with estrogen signaling pathways also being markedly enriched in this group. This finding provides novel insights for developing age-specific therapeutic strategies for breast cancer and underscore the need for further research into age-related molecular mechanisms.

Andersson et al. [65] employed ST to analyze eight HER2+ breast cancer specimens, systematically revealing spatial heterogeneity within the tumor microenvironment. Data normalization and unsupervised clustering identified region-specific molecular subpopulations, including metabolic pathway gradients (e.g., glycolysis/oxidative phosphorylation), spatially compartmentalized immunosuppressive signaling (TGF-β/IFN-γ co-localization zones), and tumor margin-enriched epithelial-mesenchymal transition (EMT) gene modules. Spatial interaction analysis demonstrated CXCL10+ M2 macrophage-derived chemotactic gradients at tumor–stroma interfaces and CXCL13/CCL19 chemokine gradients within B-T cell co-localization regions. The study innovatively quantified spatial heterogeneity through metrics assessing molecular dispersion, revealing heightened spatial constraint in EGFR/ERBB2-enriched areas versus diffuse distribution of immune checkpoint molecules (PD-L1/LAG3). This work established a novel spatial omics framework for investigating tumor ecosystem dynamics and precision immunotherapy target discovery.

Bassiouni et al. [66] employed ST to conduct a comprehensive spatial and transcriptomic analysis of TNBC tumor tissues, revealing the heterogeneity of the TME and its relationship with clinical outcomes. The study found that hypoxia-related tumor cells were significantly enriched in African American patients and associated with poorer prognosis, while immune-enriched regions were more common in Caucasian patients. Spatial distribution analysis further uncovered a spatial exclusion relationship between tumor cells and immune cells, suggesting that hypoxic regions may promote tumor progression by suppressing immune cell infiltration. This finding provides new insights into personalized treatment for TNBC and highlight potential therapeutic strategies targeting hypoxia and the tumor immune microenvironment (TIME).

In breast cancer, some special histological subtypes have high metastatic potential or recurrence tendency. Exploring their heterogeneity is crucial for understanding the pathological mechanisms. Breast phyllodes tumors (PTs) stand out as a distinct class of fibroepithelial neoplasms. They are notable for being prone to metastasis and having a high likelihood of recurrence. Li et al. [67] integrated scRNA-seq and ST to uncover the heterogeneity and spatial distribution characteristics of stromal cells in PTs. The study revealed that COL4A1/2 plays a critical role in stromal cell differentiation and is significantly enriched at the tumor–stroma interface, where it promotes invasive tumor growth through interactions with ITGA1/B1. Additionally, COL4A1/2 and CSRP1 were identified as potential diagnostic biomarkers for PTs. This discovery provides a novel molecular foundation for the precise diagnosis and treatment of breast phyllodes tumors. Invasive micropapillary carcinoma (IMPC), another distinct histological subtype of breast cancer, is marked by an extremely high lymph node metastasis rate.

Lv et al. [68] utilized ST technology and systematically revealed for the first time the diversity of gene expression, spatial heterogeneity, and the close relationship with metabolic reprogramming in IMPC of the breast. This study revealed through ST mapping that IMPC tumor regions exhibit significant metabolic heterogeneity, characterized by concurrent activation of lipid metabolism (SREBF1/FASN pathway) and glycolytic metabolism (Warburg effect). Adjacent stroma displayed gradient expression patterns based on spatial proximity, with immunoglobulin genes highly expressed in peritumoral stroma and oxidative phosphorylation pathways enriched in distant stroma. Furthermore, spatial demarcation of TIME was observed between IMPC and invasive ductal carcinoma-not otherwise specified type (IDC-NOS) regions: IMPC regions lacked T lymphocyte infiltration-mediated immune surveillance, while IDC-NOS regions formed inhibitory barriers through extracellular matrix remodeling and immune cell interactions. Beyond delineating IMPC-specific spatial expression patterns, this study identified SREBF1 and FASN as putative spatially resolved prognostic markers. This study provides new perspectives for understanding the highly aggressive biological behavior of IMPC and lay an important foundation for the development of precise diagnosis and treatment strategies for breast cancer.

Germline mutations in the BRCA1 and BRCA2 genes are well-established risk factors for distinct subtypes of breast cancer. Specifically, BRCA1 mutations are strongly associated with basal-like breast cancers, while BRCA2 mutations are more frequently linked to luminal-like breast cancer. Notably, defects in mammary epithelial cell differentiation have been observed in germline BRCA1/2 mutation carriers even prior to the onset of cancer. However, the molecular mechanisms driving these differentiation abnormalities remain poorly understood. Caputo et al. [69] utilized the NanoString GeoMx DSP platform to conduct ST analysis on breast tissues from 12 BRCA1/2 mutation carriers and non-carriers, revealing the spatial distribution and functional heterogeneity of epithelial and stromal cells. The findings indicated that BRCA1 and BRCA2 mutations exerted unique effects on gene expression in breast cancer cells: BRCA1-mutated cells exhibited a pronounced tendency for ECM degradation, specifically characterized by upregulation of matrix metalloproteinases such as MMP3 and MMP8, whereas BRCA2-mutated cells regulated ECM remodeling through integrin-mediated mechanotransduction mechanisms, such as collagen binding and cytoskeletal tension modulation. Further ST data analysis revealed that, in BRCA1/2-mutated tissues, the interaction between stromal cells and the ECM is significantly enhanced, particularly with elevated expression levels of integrins and syndecan receptors in stromal cells. Validation via immunofluorescence staining showed that the ITGA6-COL6A1 receptor–ligand pair exhibited a positive correlation in non-mutated tissues, but this correlation was lost in mutated tissues. These significant findings not only elucidate the specific regulatory mechanisms of BRCA1/2 mutations on the breast TME but also provide a solid theoretical and experimental foundation for developing precision-targeted therapies for BRCA1/2 mutation-associated breast cancer.

Phenotypic heterogeneity is a fundamental feature of biological systems, enabling populations to enhance their chances of survival under dynamically varying environmental stresses. Cancer cells exhibit significant heterogeneity along functional and molecular axes, which allows them to evade therapeutic attacks, adapt to changing environments, and ultimately drive metastasis and colonization [70]. Sahoo et al. [71] analyzed the relationship between two key axes of heterogeneity in breast cancer, EMT and luminal-basal (lineage) plasticity, by integrating scRNA-seq, bulk, and ST data. The study revealed that the luminal–epithelial association and the basal-partial EMT (pEMT) association are strongly positively correlated across breast cancer samples and model systems. Basal-like breast cancers exhibited higher EMT heterogeneity and were often in a hybrid E/M state, a dynamic phenotype in which tumor cells co-express both epithelial (E) and mesenchymal (M) characteristic markers during metastasis, which is associated with poorer patient survival and treatment response. In contrast, luminal breast cancers primarily displayed an epithelial phenotype and were more homogeneous, although luminal tumors with low ER expression may exhibit more basal-like characteristics. Additionally, a mutual driving relationship was observed between EMT and tamoxifen resistance, suggesting that tamoxifen resistance may regulate lineage plasticity (e.g., luminal-to-basal switching). This finding provides new insights into understanding phenotypic heterogeneity in breast cancer and its clinical implications. Intra-tumoral phenotypic heterogeneity also promotes tumor lymph node metastasis.

Chung et al. [72] employed DSP and bioinformatics to compare ST profiles between eight lymph node-positive and lymph node-negative breast cancer patients. The study revealed that, in the epithelial compartment of the lymph node-positive group, the NR4A1 and JUN genes were significantly upregulated, and genes associated with myeloid differentiation, mononuclear cell differentiation, and hematopoietic regulation were notably enriched. Furthermore, gene set enrichment analysis (GSEA) demonstrated significant enrichment of gene sets related to the regulation of inflammatory cytokines in both the epithelial and stromal compartments of the lymph node-positive group. This finding highlights substantial differences in gene expression and spatial transcriptional activity between lymph node-positive and lymph node-negative breast cancers, underscoring the importance of developing personalized treatment strategies for breast cancer patients with lymph node metastasis. Wu et al. [73] integrated scRNA-seq and ST to decode breast cancer heterogeneity. Their high-resolution cellular taxonomy identified nine major cell types with multiple functional states, revealing universal spatial heterogeneity across epithelial, immune, and stromal compartments. Spatial mapping uncovered mutually exclusive zones in basal-like tumors: EMT-enriched invasive fronts versus proliferative cores. Nine prognostic ecotypes were defined by cell co-localization patterns, with 32% of luminal tumors harboring basal/HER2-like subclones detected via scSubtype algorithm, potentially driving therapy resistance. Metabolic gradients showed lipid metabolism upregulation near tumor margins versus oxidative phosphorylation dominance in distal stroma. Immunosuppressive niches formed by TREM2+ macrophages adjacent to PD-1+ lymphocytes correlated with poor outcomes. CAF exhibited spatial segregation across 3–5 plastic states, suggesting microenvironment-driven differentiation. This multidimensional spatial framework advances precision oncology beyond conventional immune-based classification.

Table 2 summarizes the applications of spatial transcriptomics in deciphering breast cancer heterogeneity.

### 2.2. Characterizing Tumor Microenvironment in Breast Cancer

Intratumoral heterogeneity highlights the differences between cells or cell subsets within TME, which can profoundly influence tumor initiation, progression, invasion, metastasis, and drug sensitivity [74]. The TME is composed of a heterogeneous populations of tumor, immune, and stromal cells. It can be categorized into two distinct types: an TIME dominated by immune cells and a non-immune TME primarily consisting of fibroblasts. Currently, cancer research and treatment strategies have shifted from a tumor-centric model to a tumor microenvironment-centric model, reflecting the growing recognition of the TME’s critical role in cancer biology [75].

Table 3 summarizes the applications of ST in investigating the TME of breast cancer.

#### 2.2.1. TIME

Notably, by analyzing the spatial distribution of gene expression within TIME, ST holds the potential to unearth novel spatial prognostic markers for breast cancer. Romanens et al. [76] successfully analyzed the transcriptional profiles of distinct regions within the TME of TNBC, including the tumor core (TC), stromal tumor-infiltrating lymphocytes (sTIL), and fibroblasts, by optimizing the LCM and exome-capture RNA sequencing (ecRNA-seq) workflows for FFPE samples. ST analysis revealed significant functional differences between TC and sTIL: sTIL were enriched in pathways related to immune cell functions, such as TCR, NKT, BCR, JAK-STAT, and IL-2/3/5 signaling, while TC exhibited enrichment in pathways associated with cell cycle regulation, cell junction organization, and membrane trafficking. Additionally, the study found that intraepithelial T cells (cTIL) displayed lower immune repertoire diversity but higher clonality compared to sTIL, suggesting that cTIL may undergo clonal expansion due to recognition of specific tumor antigens. These findings underscore the critical role of ST analysis in unraveling the heterogeneity of the TME and the functional dynamics of immune cells, providing essential insights for the development of precision therapeutic strategies.

Wu et al. [77] developed a CD8+ T cell-related (CTR) score by integrating multi-omics data and machine learning algorithms, revealing its critical role in predicting breast cancer prognosis. The study found that patients in the high CTR score group had poorer prognoses and exhibited significant immunosuppressive characteristics within TME. The CTR score not only predicts patient responses to chemotherapy and immunotherapy but also provides new insights for the development of personalized treatment strategies. Future research is needed to further validate the clinical utility of the CTR score and to explore the molecular mechanisms of related genes in breast cancer progression.

Yu et al. [78] found that high expression of DNA damage-inducible transcript 3 (DDIT3) promotes the formation of an immunosuppressive TME by increasing the infiltration of regulatory T cells (Tregs) and M2-type macrophages while reducing naïve B cells and resting memory CD4+ T cells. Additionally, DDIT3 is highly expressed in monocytes/macrophages, fibroblasts, and malignant cells. In monocytes/macrophages, DDIT3 may enhance immunosuppression by promoting M2-type polarization; in fibroblasts, it may support tumor growth by regulating extracellular matrix remodeling and growth factor secretion; and in malignant cells, DDIT3 may drive proliferation, inhibit apoptosis, and enhance invasiveness. ST confirmed that DDIT3 co-localizes with markers of malignancy, fibroblasts, and immune cells, highlighting its multifaceted role in tumor progression. This finding suggests that DDIT3 may serve as a potential target for immunotherapy and a prognostic biomarker, warranting further research into its mechanisms and clinical applications.

Tzeng et al. [79] demonstrated that MiCU1/2 regulates immune cell infiltration and function in the TME. High MiCU1/2 expression correlates with increased infiltration of M2-type tumor-associated macrophages (TAMs), which promote tumor progression, angiogenesis, and immune evasion via immunosuppressive cytokines like IL-10 and TGF-β. MiCU1/2 may enhance TAM activity, reinforcing an immunosuppressive microenvironment and aiding tumor immune escape. In breast cancer, the upregulation of MiCU1/2 is linked to poor prognosis, reduced survival, and increased immune cell (particularly macrophages) infiltration, suggesting its role in immunosuppression. Additionally, MiCU1/2 may influence immune checkpoint recognition. ST analysis revealed MiCU1/2’s high expression in tumors and immune cells, highlighting its complex spatial distribution. This study positions MiCU1/2 as a potential prognostic biomarker and immunotherapy target.

To explore the breast cancer microenvironment, Liu [80] used GeoMx DSP technology to examine transcripts from 107 regions of interest (ROIs) in 65 untreated breast cancer samples. The study uncovered significant spatial heterogeneity in marker gene expression among tumor-rich, immune-rich, and normal epithelial regions. A total of 55 prognostic markers were identified in tumor-rich regions and 15 in immune-rich regions. Tumor-rich areas showed higher levels of follicular helper T cells, resting dendritic cells (DCs), and plasma cells but fewer resting memory CD4+ T cells and Tregs compared to immune-rich regions. The heterogeneity of human leukocyte antigen (HLA) gene families, immune checkpoints, and metabolic genes was also analyzed. Univariate Cox analysis revealed five metabolic genes linked to prognosis. Immunostaining experiments validated key findings, particularly for EMILIN2, SURF4, and LYPLA1. This study highlights spatial heterogeneity in the breast cancer TME, identifying diagnostic and prognostic markers with implications for precision oncology.

#### 2.2.2. Non-Immune TME

CAF represents one of the most prevalent cellular constituents within the non-immune TME of solid malignancies. Although their complexity was historically underappreciated, the heterogeneous nature of CAF has gained substantial recognition in contemporary oncology research.

Croizer et al. [81] employed ST and scRNA-seq to systematically investigate the spatial heterogeneity of FAP+ CAF in breast cancer and their interactions with immune and tumor cells. The study revealed that FAP+ CAF exhibited remarkable plasticity, with Detox-iCAF potentially serving as the origin of other subtypes, transitioning into ECM-myCAF and Wound-myCAF via the DPP4 and YAP1/TEAD signaling pathways, respectively, while the TGFβ2/TGFBR2 pathway plays a pivotal role in tumor cell-induced transformation. ST uncovered distinct distribution patterns of FAP+ CAF in the tumor bed, invasive margin, and peritumoral regions and identified 10 EcoCellTypes (ECTs) through unsupervised analysis. These ECTs are closely associated with breast cancer molecular subtypes and patient prognosis. For instance, ECT4 (Detox-iCAF and FOLR2+ TAM) correlated with an immune-protective microenvironment, whereas ECT9 (ECM-myCAF and TGFβ-myCAF) and ECT10 (Wound-myCAF and TREM2+ TAM) were linked to immune-suppressive microenvironments and poorer outcomes. Functional experiments demonstrated that FAP+ CAF modulate immune cell functions, such as promoting TREM2+ TAM differentiation and increasing FOXP3+ Treg populations, thereby shaping the TME. Furthermore, the composition of FAP+ CAF significantly differed between DCIS and invasive breast cancer, with high Detox-iCAF expression associated with lower recurrence risk and high TGFβ-myCAF expression indicating a higher risk of invasive recurrence. These findings provide novel insights for precision therapy and prognostic evaluation in breast cancer.

Honda et al. [82] proposed that different CAF populations could not be simply categorized as either immune-promoting or immune-suppressing cells, as their functional states were dynamically regulated through precise control of gene expression. The study first constructed a single-cell atlas of breast cancer, integrating data from two previously published studies, which included detailed information from 26 breast cancer samples and over 18,000 CAF. Through ST analysis, the study revealed significant spatial heterogeneity in the distribution of CAF subpopulations within tumor tissues: ECM-myCAF, Wound-myCAF, and TGFβ-myCAF were predominantly enriched in regions with high TGF-β signaling, while Detox-iCAF and IL-iCAF were mainly distributed in the tumor periphery. Further spatial gene ontology (GO) analysis uncovered the spatial distribution patterns of ECM remodeling and immune regulation-related functions within the TME. Although the TGF-β signaling pathway is generally considered to suppress tumor immune responses, the study found that certain tumor regions with high TGF-β signaling also exhibited significant CD8+ T cell infiltration, indicating a complex regulatory relationship between TGF-β signaling and immune cell infiltration. The study also observed that CD8+ T cells and macrophages were primarily located in tumor regions with low proliferative potential, while being less prevalent in highly proliferative areas. Through single-cell data spatial mapping, the study further demonstrated that the spatial distribution of ECM-myCAF, Wound-myCAF, and TGFβ-myCAF closely aligned with TGF-β signaling, while EMILIN1, an inhibitor of TGF-β signaling, was significantly associated with CD8+ T cell enrichment in regions where it was highly expressed. Immunofluorescence and immunohistochemical analyses confirmed the positive correlation between EMILIN1 expression in CAF and CD8+ T cell infiltration, and breast cancer patients with high EMILIN1 expression showed significantly improved survival rates. These findings suggest that CAF plays a crucial role in modulating the TIME through the regulation of TGF-β signaling, and EMILIN1 may serve as a potential prognostic marker and therapeutic target, offering new directions for precision therapy in breast cancer.

Ma et al. [83] revealed that, although the proportions of CAF subtypes vary across different cancer types, iCAF and mCAF consistently emerged as the predominant subtypes in all common cancer types, and their presence is also observed in rare cancer types such as EMC and MEC. The study further identified that pCAF exhibited heightened activity in IFN-I (Type I interferon) production, suggesting their potential as a target for combination immunotherapy. Additionally, the research uncovered the metabolic diversity among CAF subpopulations: mCAF was enriched in fatty acid biosynthesis, pCAF in the TCA cycle, and meCAF in glycolysis and amino acid metabolism, indicating that therapeutic strategies targeting CAF metabolism must account for subtype-specific characteristics. The study also elucidated the pericyte-to-iCAF transition (PFT) pathway, suggesting that pericytes may contribute to the formation of an immunosuppressive microenvironment and ECM remodeling by transforming into iCAF. Spatial analysis demonstrated that CAF subpopulations exhibited closer spatial proximity to each other compared to other cell types. Specifically, interactions between mCAF and EC were found to promote angiogenesis within the TME, while the co-localization of iCAF with CD8+ T cells underscored their critical role in fostering an immunosuppressive microenvironment. Furthermore, analysis of data from breast cancer patients undergoing anti-PD-1 therapy revealed that such treatment enhanced the ability of iCAF to promote immunosuppression. Notably, an iCAF score constructed based on iCAF marker genes showed a significant correlation with immunotherapy response in melanoma patients, highlighting the potential clinical value of combining iCAF-targeted interventions with anti-PD-1 therapy.

#### 2.2.3. Spatial Interaction Mechanisms Among Cells in the TME

Within the TME, tumor, immune, and stromal cells interact spatially. This crosstalk forms a dynamic network that can either promote or inhibit tumor growth, invasion, and metastasis.

Chen et al. [84] utilized ST to deeply analyze the spatial interactions between tumor cells and immune cells (such as macrophages) as well as stromal cells within the TME of TNBC. ST analysis identified spatially restricted tumor–macrophage interactions mediated by macrophage migration inhibitory factor (MIF). This ligand–receptor axis drove M2-like polarization, establishing an immunosuppressive niche that facilitated tumor progression. ST unveiled the spatial heterogeneity of the TME, showing that epithelial cells (tumor cells) and MIF are in close spatial proximity within tumor tissues, further supporting the critical role of MIF in regulating macrophage polarization and TME remodeling. This provides new insights into understanding the complex interactions within the TME. Additionally, the study found that TNBC tumor tissues with high MIF expression exhibited a significant increase in M2-type macrophages, and high MIF expression was associated with worse clinicopathological stages and patient prognosis. This finding indicates that the spatial distribution and functional state of MIF within the TME have a significant impact on tumor progression.

Zhu et al. [85] employed integrated multi-omics analyses to comprehensively investigate the spatial interactions between tumor cells and immune cells (such as CD8+ T cells) as well as stromal cells within the tumor TME of TNBC, with a particular focus on the critical role of the OTUD4/CD73 proteolytic axis in tumor immune evasion. The study revealed that OTUD4 stabilizes CD73 through deubiquitination, thereby suppressing the cytotoxic function of CD8+ T cells and promoting tumor immune escape. TGF-β signaling further enhances this immunosuppressive effect by regulating the OTUD4/CD73 axis. ST analysis uncovered the spatial distribution of CD73 within tumor cells and its interaction with OTUD4, demonstrating that the membrane localization and intracellular reservoir of CD73 are dynamically regulated by a balance between ubiquitination (mediated by TRIM21) and deubiquitination (mediated by OTUD4). Additionally, the study developed a small-molecule inhibitor, ST80, which specifically disrupted the interaction between OTUD4 and CD73, promoting CD73 ubiquitination and degradation, thereby restoring CD8+ T cell function. The combination of ST80 with anti-PD-L1 therapy significantly enhanced the therapeutic efficacy against TNBC tumors, effectively inhibiting tumor growth even in cases with high expression of OTUD4 and CD73. These findings elucidate the spatial regulatory mechanisms of the OTUD4/CD73 axis within the TME and provide a novel therapeutic strategy to improve the efficacy of immunotherapy for TNBC.

Janesick et al. [86] integrated scRNA-seq with ST technologies (Visium CytAssist and Xenium) to investigate the spatial interactions among tumor cells, immune cells, and stromal cells within the TME of breast cancer. The study revealed significant tumor heterogeneity, particularly in the distribution of myoepithelial cells between ductal carcinoma in situ (DCIS) and invasive tumor regions. DCIS regions were enriched with ACTA2+ and KRT15+ myoepithelial cells, while these cells were entirely absent in invasive areas. Xenium in situ analysis provided high-resolution spatial gene expression data, identifying a unique population of “boundary cells” co-expressing tumor markers (e.g., ERBB2 and ABCC11) and myoepithelial markers (e.g., MYLK and DST) in the transitional zone from DCIS to the invasive tumor, suggesting their potential role in tumor progression. The discovery of “boundary cells” and the detailed mapping of interactions between immune and stromal cells provide a foundation for developing precision therapeutic strategies targeting the TME.

Tzeng et al. [87] utilized scRNA-seq and ST technologies to deeply investigate the TME of breast cancer. The study revealed that the high expression of the LSM1 gene is closely associated with tumor progression, particularly showing significant upregulation in DCIS and invasive tumor regions. The elevated expression of LSM1 was closely linked to tumor cell energy metabolism, including oxidative phosphorylation and the PI3K/AKT/mTOR signaling pathway, and significantly impacted mitochondrial function and cellular respiration. The study of ST further elucidated the distribution and functional states of immune cells, such as macrophages, within the TME. M2-type macrophages were notably increased in tumor regions with high LSM1 expression, suggesting that LSM1 may promote the formation of an immunosuppressive TME by regulating macrophage polarization and function. Additionally, the study found a positive correlation between LSM1 expression and macrophage markers CD163 and CXCR4, indicating that LSM1 may influence tumor immune evasion and progression by modulating macrophage infiltration and activity. Furthermore, by using ST, the study demonstrated significant overexpression of metabolism-related genes (e.g., HK2, PDHA1, and CS) in tumor regions, further supporting the critical role of LSM1 in regulating tumor energy metabolism. Consistent with Janesick’s findings [86], Tzeng et al. also validated the existence of “boundary cells”, providing new insights into the spatial interactions between tumor cells, immune cells, and stromal cells and highlighting the important role of LSM1 in the breast cancer TME.

**Table 3:** Application of ST in TME of breast cancer.

Classification		Technique	Cell Line/Marker	Main Findings	References
TIME	T cells	ST-FFPE; GeoMx DSP; 10×Visium	TILs, CD8+ T cells, Tregs, Follicular helper T cells	• High intra-epithelial TILs clonality reflect tumor-antigen specific expansion and predicts favorable immunotherapy response in TNBC. • The high CTR score reflects an immunosuppressive TME characterized by suppressed CD8+ T cell function. • High DDIT3 expression increases Tregs infiltration, promoting an immunosuppressive TME. • Follicular helper T cells are more abundant in tumor cell-enriched regions. • The function of CD8+ T cells is regulated by the OTUD4/CD73 axis, suppressing cytotoxicity and promoting tumor immune escape.	[76,77,78,80,85]
	Macrophages	Slide-seq; 10×Visium	M2-type TAMs	• DDIT3 enhances immunosuppression by promoting M2-type polarization. • M2-type TAMs promote tumor progression and immune escape via IL-10 and TGF-β secretion. • High MiCU1/2 expression increases M2-type TAMs infiltration, enhancing immunosuppression. • MIF drives M2-type TAMs polarization, shaping an immunosuppressive TME. • LSM1 may influence tumor immune evasion and progression by regulating macrophage polarization and function.	[78,79,84,87]
	B cells	GeoMx DSP	Naïve B cells, Plasma cells	• High DDIT3 expression reduces naïve B cells, promoting an immunosuppressive TME. • Plasma cells are more abundant in tumor-cell-enriched regions.	[78,80]
	DCs	GeoMx DSP	Resting DCs	Resting DCs are more abundant in tumor cell-enriched regions.	[80]
	Immune checkpoints	10×Visium	CD73, PD-L1	• The OTUD4/CD73 axis regulates CD8+ T cell function by modulating CD73 ubiquitination. • Anti-PD-L1 therapy combined with ST80 (a CD73 inhibitor) significantly enhances TNBC treatment efficacy.	[85]
Non- immune TME	Metabolism-related genes	10×Visium	LSM1, HK2, PDHA1, CS	• High LSM1 expression is linked to tumor energy metabolism, significantly impacting mitochondrial function and cellular respiration. • Metabolic genes in tumor-cell-enriched regions are associated with prognosis.	[86,87]
	CAFs	10×Visium; Slide-seq; ST-FFPE	Detox-iCAF, ECM-myCAF, Wound-myCAF, TGFβ-myCAF, pCAF, mCAF, meCAF	• DDIT3 supports tumor growth by regulating extracellular matrix remodeling and growth factor secretion. • CAFs exhibit significant heterogeneity; Detox-iCAF may transform into other subtypes via DPP4 and YAP1/TEAD signaling. • mCAF enriches fatty acid synthesis, pCAF enriches the TCA cycle, and meCAF enriches glycolysis and amino acid metabolism. • iCAF co-localizes with CD8+ T cells, promoting an immunosuppressive TME.	[78,81,82,83]
	Tumor cells	Slide-seq; 10×Visium	Malignant cells, Boundary cells	• DDIT3 drives proliferation, inhibits apoptosis, and enhances invasiveness in malignant cells. • Boundary cells in the DCIS-to-invasive transition zone express tumor markers and myoepithelial markers, potentially contributing to tumor progression.	[78,86,87]
	ECM	10× Visium; ST-FFPE	ECM-remodeling-related genes	• DDIT3 regulates ECM remodeling and growth factor secretion in fibroblasts, supporting tumor growth. • CAFs modulate immunosuppression through ECM remodeling.	[82,83]
	ECs	ST-FFPE	Vascular endothelial cells	mCAF interacts with endothelial cells to promote angiogenesis in the TME.	[83]

Abbreviations: ST-FFPE, spatial transcriptomics for formalin-fixed paraffin-embedded; TILs, tumor-infiltrating lymphocytes; CTR, CD8+ T-cell-related; TME, tumor microenvironment; TIME, tumor immune microenvironment; TAMs, M2-type tumor-associated macrophages; DCs, dendritic cells; CAFs, cancer-associated fibroblasts; DCIS, ductal carcinoma in situ; ECs, endothelial cells; ECM, extracellular matrix.

### 2.3. Spatial Dynamic Alterations During the Progression and Metastasis of Breast Cancer

Cancers affect host cells in various ways to ensure their survival and evade the host immune system. Exploring the dynamic changes in the TME and the interactions between cells during the progression stage of breast cancer can comprehensively and profoundly reveal the intricate biological processes underlying the progression of breast cancer.

Mao et al. [88] integrated single-cell transcriptomics and ST to track gene expression changes and immune cell dynamics during breast tumor progression. The study revealed that immune cell infiltration was significantly higher in breast tumor tissues compared to normal tissues, with epithelial cells and immune cells closely co-localized in space, suggesting their interactions may drive tumor progression. This finding underscores the importance of immune cell dynamics in cancer progression.

Cai et al. [89] integrated scRNA-seq and ST data to investigate the dynamic changes in the TME and spatial organization during the progression of breast cancer from DCIS to invasive ductal carcinoma (IDC). The study revealed that, as the tumor advanced, there was a significant increase in copy number variation (CNV) and the activity of tumor-related signaling pathways, such as TGF-β, Wnt, and STAT3, leading to enhanced proliferation and invasiveness of tumor cells. Additionally, the proportion of Treg cells and CXCL13+ T cells was elevated in the IDC stage, and tumor cells frequently interacted with T cells through co-inhibitory axes like NECTIN2/TIGIT, resulting in T cell exhaustion and the establishment of an immunosuppressive TME. ST data further confirmed the spatial co-localization of tumor and immune cells, as well as the expression of co-inhibitory ligand–receptor pairs, such as SPP1/CD44 and MIF/CD74, highlighting the mechanism by which tumor cells promote immune escape through the expression of co-inhibitory factors. This finding provides critical insights into the mechanisms underlying the formation of an immunosuppressive TME during breast cancer progression.

The gene expression alterations and spatial dynamics during the progression of distinct molecular subtypes of breast cancer might exhibit specificity. Xu et al. [90] integrated scRNA-seq and ST data to systematically characterize the TME of ER+ breast cancer, tracking gene expression changes and spatial dynamics during tumor progression. The study revealed that, as the disease advanced, the proportion of epithelial cells (e.g., luminal and cycling cells) significantly increased in ER+ tumors, with immune cells (e.g., T cells and macrophages) enriched around epithelial cells, suggesting that their physical interactions with tumor cells may drive tumor proliferation. Through CNV analysis, the study identified malignant cell subpopulations in ER+ tumors and uncovered their spatial distribution and functional heterogeneity at the invasive front. Malignant cells interacted with macrophages and T cells via ligand–receptor pairs such as MIF-CD74 and MDK-LRP1, fostering an immunosuppressive TME that promotes tumor progression. Furthermore, the study constructed an intercellular gene regulatory network, elucidating molecular mechanisms by which macrophages and T cells regulate malignant cell proliferation and invasion through signaling pathways such as AREG–ERBB3–FOXA1–ESR1. ST data further validated the spatial co-localization of these ligand–receptor pairs and their functional significance in the TME. The robustness of key signaling molecules (e.g., EZH2, FOXA1, and ESR1) was confirmed using an independent dataset, providing novel insights into the regulatory mechanisms of the TIME in ER+ breast cancer.

In addition to the dynamic TME of the primary tumor, the cellular adaptability and TME remodeling during tumor metastasis are also worthy of attention. Liu et al. [91] discovered that early disseminated tumor cells (EDCs) are enriched at the border of primary tumors and undergo a metabolic shift between glycolysis and oxidative phosphorylation (OXPHOS) during the dissemination process. EDCs initially exhibit upregulated OXPHOS but transition to enhanced glycolysis upon colonization, reflecting a more aggressive phenotype. The study also revealed highly active interactions between EDCs and immune cells via the MIF pathway, highlighting its critical role in the TME. Furthermore, by analyzing paired primary tumor and metastatic lymph node samples, the metabolic characteristics of metastatic tumor cells were validated. Despite limitations in sample size and spatial heterogeneity, the integration of scRNA-seq and ST data elucidated the dynamic metabolic evolution of tumor cells during early dissemination and their interactions with the microenvironment, offering potential new directions for prognostic assessment and therapeutic strategies in breast cancer.

Maeshima et al. [92] utilized ST and imaging mass spectrometry to unveil significant spatial heterogeneity in the TIME of lymph nodes in metastatic breast cancer, particularly highlighting the dynamic changes in CD169+ macrophages. The study revealed that CD169+ macrophages, crucial players in early anti-tumor immunity, were progressively suppressed and eliminated in metastatic breast cancer. This suppression, potentially mediated through direct or indirect mechanisms, led to the impairment of anti-tumor immune responses, thereby facilitating the spread of cancer cells within the lymph nodes. The research initially employed laser microdissection and RNA sequencing to compare gene expression differences between non-metastatic and metastatic lymph nodes in breast cancer patients, identifying significant downregulation of macrophage-related genes (e.g., SIGLEC1, MARCO, and AIM) in metastatic lymph nodes. Further spatial transcriptomic analysis confirmed a marked reduction in the number of macrophages, especially CD169+ macrophages, in metastatic lymph nodes, while the quantities of other immune cells such as B cells and T cells remained relatively stable. Imaging mass spectrometry further validated these findings, demonstrating a significant decrease in the density of CD169+ macrophages in metastatic lymph nodes, which negatively correlated with the pathological staging (pN classification) of breast cancer. The study also found that the reduction in CD169+ macrophages was a common phenomenon in the process of breast cancer metastasis, observed across luminal, HER2+, and TNBC subtypes. Moreover, the decrease in CD169+ macrophages may have preceded other immune cell abnormalities and systemic inflammation, suggesting its early role in breast cancer immune evasion.

Table 4 summarizes the applications of ST in investigating spatiotemporal dynamics during breast cancer progression and metastasis.

### 2.4. Advancing Precision Therapy in Breast Cancer

The treatment of breast cancer encompasses a diverse array of modalities, such as chemotherapy, neoadjuvant therapy (NAT), targeted therapy, immunotherapy, etc. Nevertheless, the pronounced heterogeneity inherent in breast cancer gives rise to a highly intricate TME. This complexity poses formidable challenges to treatment strategies. In this context, the ST technology emerges as a powerful tool, offering a novel and unique perspective for delving deep into the therapeutic mechanisms underlying breast cancer treatment and the complex phenomenon of drug resistance.

#### 2.4.1. Chemotherapy

Kulasinghe et al. [93] utilized Nanostring GeoMx DSP(NanoString Technologies, Seattle, WA, USA) combined with clinical follow-up data to reveal that the protein expression profiles in the tumor (PanCK+) and stromal (PanCK-) regions significantly predict chemotherapy response. In chemotherapy-responsive patients, the expression of estrogen receptor alpha (ERα) in the stromal region was significantly elevated, while 41BB and MART1 expressions were reduced. In the tumor regions, GZMA, STING, and fibronectin expression were upregulated, whereas CD80 expression was downregulated. These differentially expressed proteins were significantly associated with overall survival (OS), with high expressions of ERα and GITR correlating with better prognosis, while high expression of MART1 indicated poorer outcomes. Additionally, multivariate analysis identified multi-protein signatures associated with chemotherapy response in both tumor and stromal regions: high expression of NF1 in the tumor region and ERα in the stromal region were significantly linked to chemotherapy response. The study also found that high expression of PD-L1, FOXP3, and GITR in the tumor regions was associated with longer OS, suggesting that Tregs and GITR-expressing TILs may play an active role in modulating immune activity. This finding provides new insights for the precision treatment and prognostic assessment of TNBC.

#### 2.4.2. NAT

In recent years, NAT for breast cancer has received increasing attention, among which neoadjuvant chemotherapy (NAC) is a common way. Donati [94] utilized ST technology to analyze tumor tissues from TNBC patients before and after NAC, revealing spatial expression patterns and gene signatures associated with treatment response. The study found that the distribution and functional states of immune cells within the TME significantly influence the efficacy of NAC. Pathological complete response (pCR) patients exhibited expanded TIL populations, especially CD8+ and CD4+ T cells, indicating their cytotoxic role in tumor elimination. Concurrently, IFN-pathway-related genes (e.g., STAT1, STAT2, and IRF9) were markedly upregulated in both tumor regions (CK+ AOIs) and stromal regions (CK- AOIs), indicating that the activation of the IFN signaling pathway is associated with favorable responses to NAC. In contrast, in non-pathological complete response (pNR) patients, genes related to angiogenesis (e.g., VEGFA and ANGPT1) and oxidative metabolism were significantly upregulated in tumor regions, suggesting that enhanced angiogenesis and oxidative metabolism may contribute to chemotherapy resistance. Additionally, pNR patients exhibited an increase in immunosuppressive cells (e.g., mast cells) and a reduction in effector T cells and mature dendritic cells (mDCs), creating an immunosuppressive TME. Furthermore, low expression of HLA class I molecules in pNR patients implied impaired antigen presentation, potentially leading to immune evasion. In pCR patients, immune-activation-related genes (e.g., CXCL9, CD68, CD3, and CD4) were significantly upregulated, further supporting the association between enhanced immune response and favorable NAC outcomes. Independent validation cohorts also confirmed the correlation between immune activation and positive NAC responses, providing new insights into the mechanisms underlying TNBC treatment responses.

On the basis of clarifying the dynamic changes of TIME under the action of NAC, recent research has further focused on the synergistic mechanism of combined radiotherapy and immunotherapy in NAT. Through the analysis of tumor tissues from TNBC patients before and after NAT with pembrolizumab and localized radiotherapy (RT), Shiao et al. [95] utilized scRNA-seq and spatial analysis techniques to uncover the spatial heterogeneity of cellular communities within the TME and their dynamic changes during treatment. The study revealed that the TME of TNBC patients exhibited significant spatial heterogeneity, characterized by 12 recurrent cellular “districts”. These districts were composed of diverse immune cells, stromal cells, and epithelial cells, each playing distinct roles in therapeutic response. For instance, T cell-enriched districts and regions dominated by antigen-presenting macrophages were closely associated with treatment efficacy. The study demonstrated that, in patients receiving combined PD-1 inhibitor and localized RT, the TME of responders underwent substantial remodeling, marked by an increase in CD8+ T cells and antigen-presenting macrophages, alongside a reduction in epithelial cells. Spatial heterogeneity analysis further indicated that B cell-dominated districts were more prominent in responders at baseline, while T cell-enriched districts showed a significant increase in both proportion and density post-treatment. Additionally, the study identified two distinct response subtypes: the R1 group, which exhibited high immune infiltration at baseline and displayed typical immune checkpoint inhibitor (ICI) response characteristics, and the R2 group, which had low immune infiltration at baseline but demonstrated significant T cell expansion and TME remodeling following combination therapy, suggesting that RT played a pivotal role in enhancing immune responses. These findings not only elucidated the complex spatial heterogeneity of the TNBC TME but also provided a critical foundation for designing future combination therapies integrating immunotherapy and RT. This approach may help optimize treatment strategies and reduce the toxicity associated with chemotherapy.

#### 2.4.3. Immunotherapy

Immune checkpoint blockers (ICBs) have revolutionized cancer treatment strategies, representing a paradigm-shift in the fight against cancer. Wu et al. [96] combined scRNA-seq and ST to define KLF5’s role in metastatic lymph nodes and its immunosuppressive mechanisms within the TIME. The study revealed that KLF5 deletion significantly slowed tumor growth and increased intratumoral CD8+ T cell infiltration, indicating that KLF5 promoted tumor progression by suppressing CD8+ T cell infiltration. ScRNA-seq and ST analyses further confirmed that KLF5-low tumors exhibited a marked increase in CD4+ and CD8+ T cells, with denser spatial distribution and enhanced proliferative and functional activity. KLF5 was shown to promote PGE2 production by transcriptionally activating the COX2 gene, thereby inhibiting CD8+ T cell infiltration. KLF5/Cox2 axis inhibition synergized with anti-PD1 therapy, highlighting the therapeutic potential of targeting KLF5/COX2/PGE2 in TNBC. These findings provided critical insights into the mechanisms of tumor immune evasion and offered a foundation for developing targeted therapeutic strategies.

Tashireva et al. [97] utilized ST technology (Visium 10×) combined with multiplex immunofluorescence staining to systematically analyze the spatial heterogeneity of PD-L1-negative and PD-L1-positive TNBC tissues and its potential impact on immunotherapy response. The study revealed that, although the TME of both PD-L1-negative and PD-L1-positive patients exhibited high levels of TILs, there were significant differences in their spatial distribution and functional states. Through ST analysis, the study identified 12 recurrent cellular “districts”, composed of diverse immune cells, stromal cells, and epithelial cells, each playing distinct roles in therapeutic response. For instance, PD-L1-positive tumors showed a significantly higher proportion of M2 macrophages and CD4+ naïve T cells compared to PD-L1-negative tumors, and these cells were spatially closer to tumor cells, suggesting their potential influence on immune responses through paracrine mechanisms. Additionally, PD-L1-positive tumors demonstrated significantly elevated expression levels of HLA class II genes (e.g., HLA-DRA and HLA-DPA1) and TGFB1, along with a greater number of CD8A and CD4 expression spots, indicating a more active TIME. In contrast, PD-L1-negative tumors exhibited higher expression of antigen-presentation-related genes but lacked activation of effector immune responses, suggesting potential immune suppression. The study also found that cells co-expressing PD-L1 and PD1 (e.g., M1 macrophages and T lymphocytes) were more common in PD-L1-negative tumors but less frequent in PD-L1-positive tumors, which may explain the lack of response to immune checkpoint inhibitors (ICI) in some patients. These findings not only elucidate the complex spatial heterogeneity of the TNBC TME but also provide a critical foundation for developing more precise predictive biomarkers and optimizing immunotherapy strategies in the future.

It is worth noting that, in recent years, the role of macrophages in immunotherapy has gradually been emphasized. Through scRNA-seq and ST analyses, Xue et al. [98] revealed that macrophages exhibit the highest tryptophan metabolic activity across various tumor types, and this activity is positively correlated with M1 polarization. Spatial heterogeneity analysis demonstrated that regions enriched with M1 macrophages in breast cancer are consistently associated with elevated tryptophan metabolic activity, indicating a spatially specific relationship between tryptophan metabolism and M1 polarization. Weighted gene co-expression network analysis (WGCNA) further showed that gene modules related to tryptophan metabolism are enriched in immune-related pathways, and breast cancer patients in the high tryptophan metabolism group exhibited higher immune scores and increased infiltration of immune-activated cells, such as M1 macrophages and CD8+ T cells. Additionally, patients expected to respond to immunotherapy displayed significantly higher tryptophan metabolic activity, particularly in CCR2, CCL2, CX3CR1, and MMP9 macrophage subtypes. Experimental validation confirmed that tryptophan metabolism promotes M1 polarization of macrophages, while inhibitors of tryptophan metabolic enzymes suppress this process. These findings not only highlight the critical role of tryptophan metabolism in macrophage polarization and the TIME but also provide a theoretical foundation for its clinical application in breast cancer immunotherapy.

#### 2.4.4. Combination of Immunotherapy and Other Treatment Strategies

Given the pivotal role that macrophages play in immune regulation, a series of combined treatment strategies that target macrophages and integrate immunotherapy have been initiated. Choi et al. [99] employed ST and scRNA-seq technologies to systematically evaluate paclitaxel’s (PTX) antitumor efficacy in TNBC, elucidating its relationship with TLR4 signaling and TAM-mediated cross-presentation while characterizing TME spatial heterogeneity. The study revealed that PTX acted as a TLR4 agonist, primarily targeting TAMs with high TLR4 expression. ST analysis demonstrated that TLR4 expression was strongly correlated with the spatial distribution of myeloid cells in the TME, particularly in macrophages. In TNBC mouse models, TAMs exhibited the highest TLR4 expression, indicating their sensitivity to PTX. GSEA further showed that TLR4 signaling and cross-presentation pathways were significantly upregulated in TAMs from PTX responders, accompanied by enhanced interferon (IFN)-α and IFN-γ responses. These findings unveiled the spatial heterogeneity of TAMs within the TME and their critical role in mediating PTX-induced antitumor immunity through TLR4-dependent mechanisms. The study also demonstrated that PTX enhanced the cross-presentation capacity of TAMs, thereby promoting CD8+ T cell activation and antitumor efficacy, particularly when combined with PD-1 blockade. This combination therapy exhibited superior antitumor effects in TNBC models, underscoring the potential of targeting TAMs and TLR4 signaling to improve immunotherapy outcomes in TNBC. The results not only revealed the spatial heterogeneity of cellular populations within the TME but also provided a critical foundation for developing precision treatment strategies based on spatial heterogeneity in the future.

O’Connell et al. [100] analyzed peripheral blood and paired tumor biopsy samples from patients treated with eganelisib (a PI3K-γ inhibitor) in combination with atezolizumab (a PD-L1 inhibitor) and nab-paclitaxel in the MARIO-3 clinical trial, revealing systemic immune activation and spatial heterogeneity within the TME following treatment. Peripheral blood analysis demonstrated upregulation of immune-activating cytokines (e.g., IFNG, CXCL9, and CXCL10) and downregulation of immunosuppressive cytokines (e.g., CCL22 and TGFA), alongside a reduction in monocytic myeloid-derived suppressor cells (mMDSCs) and increased proliferation of memory T cells. Immunofluorescence and DSP of tumor biopsies further uncovered spatial heterogeneity within the TME: macrophage activation and T cell infiltration were observed in both PD-L1-positive and PD-L1-negative tumors, with significant downregulation of ECM-related genes in PD-L1-negative tumors, suggesting that the treatment potentially reversed the immunosuppressive TME. Additionally, DSP analysis revealed upregulation of immune-activation-related genes (e.g., CXCL9 and CCL5) and downregulation of ECM-organization-related genes (e.g., SPP1 and FN1) in leukocyte-enriched regions, particularly in PD-L1-negative tumors. These changes correlated with clinical outcomes, as patients exhibiting TAM reprogramming and immune activation signatures had longer progression-free survival (PFS). The study supports the role of eganelisib in the triple therapy, particularly its potential to enhance antitumor immune responses through TAM reprogramming and ECM remodeling in PD-L1-negative tumors.

#### 2.4.5. Chemotherapy Resistance

Despite the achievements attained in breast cancer treatment, the issue of drug resistance persists. Through ST and scRNA-seq analysis, Hu et al. [101] revealed a close association between PTX treatment failure and the spatial heterogeneity of EC within the TME. The study found that the immunoregulatory endothelial subpopulation EC_sub2 significantly increased in non-responders (NR) following PTX treatment. This subpopulation highly expressed immune regulatory genes such as HLA-C and enhanced immunosuppressive functions via the IFNγ signaling pathway. Spatial analysis demonstrated that EC_sub2 was in close proximity to exhausted CD8+ T cells, suggesting that EC may suppress T cell function through the PD-L1/PD-1 axis. Additionally, the TNF-TNFR2 signaling pathway inhibited glycolytic metabolism in EC by activating NF-κB, further promoting PD-L1 expression and enhancing the immunosuppressive properties of EC. Spatial co-localization analysis confirmed that TNFR2+ EC were highly aggregated with exhausted CD8+ T cells, indicating a critical role of TNFR2 in EC-mediated T cell exhaustion. Blocking TNFR2 signaling significantly improved PTX efficacy, reducing PD-L1 expression in EC and enhancing CD8+ T cell infiltration. These findings highlight the spatial heterogeneity of EC within the TME and their crucial role in PTX treatment resistance, providing a theoretical basis for improving PTX efficacy through combination therapy with TNFR2 blockade.

#### 2.4.6. Resistance to HER2-Targeted Therapies

The emergence of resistance to HER2-targeted therapies poses formidable challenges to the treatment of HER2+ breast cancer. Li et al. [102] conducted RNA sequencing on 292 pre- and post-treatment tissue samples from 129 patients to investigate the transcriptomic differences associated with HER2 heterogeneity and treatment response, with a particular emphasis on the impact of spatial heterogeneity. The study revealed significant transcriptomic variations between different regions within the same tumor, with spatial heterogeneity being more pronounced in ER- tumors. Specifically, principal component analysis (PCA) of 113 paired pre-treatment biopsy samples demonstrated substantial variability in transcriptomic distances between two distinct regions within the same tumor. Furthermore, spatial heterogeneity was also reflected in the spatial distribution of tumor cells, where HER2-high cells and basal-like marker (e.g., CK5)-high cells exhibited a mutually exclusive pattern within the tumor, further confirming significant molecular differences between distinct regions of the tumor. These findings suggest that spatial heterogeneity not only influences the biological behavior of tumors but may also play a critical role in treatment response, particularly in HER2-heterogeneous tumors, where such heterogeneity could contribute to treatment resistance. Through scRNA-seq and ST analyses, Zhang et al. [103] revealed the critical role of IMM2902 in HER2+ breast cancer, particularly in overcoming HER2 treatment resistance. IMM2902, a novel bispecific antibody, simultaneously targets CD47 and HER2, significantly enhancing anti-tumor immune responses by blocking the CD47/SIRPα signaling pathway and activating immune cell phagocytosis. Spatial heterogeneity analysis demonstrated that, following IMM2902 treatment, the infiltration of monocytes/macrophages, DCs, T cells, and NK cells within the TME was markedly increased. These immune cells were distributed more widely and uniformly throughout the tumor tissue, forming immune cell-enriched “hot spots”. In contrast, the control group exhibited minimal immune cell infiltration, primarily localized at the tumor periphery. IMM2902 significantly inhibited the growth of HER2+ breast cancer by enhancing immune cell infiltration and function, particularly showing robust anti-tumor efficacy in trastuzumab-resistant tumor models. Furthermore, IMM2902 promoted the secretion of CXCL9 and CXCL10 by macrophages, further recruiting T cells and NK cells and enhancing adaptive immune responses. These findings indicate that IMM2902 not only exerts direct anti-tumor effects by targeting tumor cells but also reshapes the TME to enhance immune cell infiltration and function, offering a novel therapeutic strategy for HER2+ breast cancer, especially for patients resistant to trastuzumab.

#### 2.4.7. Immune Therapy Resistance

For immune therapy resistance, Ge et al. [104] investigated intratumor heterogeneity (ITH) in TNBC, focusing on its spatial characteristics, mechanisms of therapy resistance, and intervention strategies. ITH manifested as genotypic, phenotypic, and functional diversity among subclonal tumor cells, primarily driven by genomic instability (e.g., aberrant activation of LINE-1 retrotransposons) and epigenetic dysregulation. Researchers demonstrated that the zinc finger protein ZNF689 suppressed LINE-1 transcriptional activity by recruiting the TRIM28 complex, thereby maintaining genomic stability. Conversely, ZNF689 deficiency triggered LINE-1 derepression, leading to DNA damage, chromosomal aberrations, and subclonal evolution, which exacerbated ITH and promoted therapeutic resistance. High ITH TNBC patients showed markedly lower PD-1 antibody response rates in clinical analyses. Key resistance mechanisms included the following: (1) impaired antigen presentation (downregulation of MHC-I and TAP1) that hindered tumor antigen recognition and (2) immunosuppressive TME remodeling characterized by reduced CD8+ T-cell infiltration, elevated Treg proportions, and diminished expression of cytotoxic markers IFN-γ and GZMB. Experimental validation confirmed that pharmacological inhibition of LINE-1 with the reverse transcriptase inhibitor efavirenz (EFV) reduced ITH, restored antigen presentation, and reversed PD-1 resistance. Furthermore, low ZNF689 expression strongly correlated with poor survival outcomes and immunotherapy resistance in TNBC patients, positioning it as a potential prognostic biomarker and therapeutic target. These findings established a novel “heterogeneity-targeting plus immunotherapy” combinatorial strategy for high-ITH tumors, providing a mechanistic foundation for clinical translation.

Table 5 summarizes the applications of ST in breast cancer treatment research.

## 3. Challenges and Future Directions of ST

ST revolutionizes the field by enabling the analysis of gene expression while preserving the spatial architecture of tissues, offering a powerful tool for studying tumor heterogeneity, microenvironment interactions, and disease mechanisms. However, its practical application faces significant challenges. One major limitation lies in the technical constraints of current ST methods, particularly in achieving true single-cell resolution. Issues such as optical crowding and transcript diffusion hinder gene detection efficiency [105,106], while the reliance on two-dimensional tissue sections limits the ability to capture gene expression in three-dimensional space, restricting its utility in studying complex tissues [107,108]. Additionally, the complexity of ST data analysis poses a substantial hurdle. The high-dimensional, large-scale datasets generated by ST are often plagued by noise, low signal-to-noise ratios, and non-uniform spatial information, making it difficult to extract meaningful insights. While AI tools (e.g., Starfysh v1.2.0 and DeepSTv1.1.0) show promise in analyzing spatial dynamics [109,110,111,112], their ST applications need further optimization [113].

High costs impede clinical translation [114], prompting development of computational methods to infer spatial patterns from single-cell data. For instance, Seurat integrates spatial and transcriptomic data to reconstruct tissue profiles and supports multimodal analysis [115,116,117]. AI-enhanced methods can also extract spatial information from HE-stained images, improving sample capture efficiency when combined with ST data [118,119,120].

Another challenge is the difficulty in sample preparation. High-quality ST experiments demand stringent conditions for tissue preservation and processing, as RNA degradation can occur rapidly, complicating experimental workflows [121]. Lack of standardized protocols compromises reproducibility and cross-study comparability. Moreover, the high costs associated with equipment, reagents, and labor limit the widespread adoption of ST, particularly in small- and medium-sized laboratories and clinical settings [114].

Despite these challenges, the future of ST is brimming with potential. Technological advancements are expected to drive significant improvements in resolution, detection depth, and data processing capabilities. Innovations in imaging technologies, such as super-resolution microscopy, could overcome current limitations, enabling precise single-cell or even subcellular-level gene expression mapping [122,]. The development of three-dimensional ST will provide a more comprehensive understanding of gene expression in complex tissues, while advancements in high-throughput sequencing and microfluidic technologies will enhance detection efficiency and reduce costs. Molecular preservation techniques are also being developed to address RNA degradation issues, improving data reproducibility and stability [123]. AI integration will enhance data analysis to extract biological insights from complex datasets.

In the clinical realm, ST holds immense promise for personalized therapy and prognostic evaluation. The impact of ST on breast cancer research and clinical treatment is particularly profound. By preserving tissue spatial architecture and analyzing gene expression, ST enables precise mapping of the spatial distribution and interactions among tumor cells, immune cells, and stromal cells, offering deep insights into the molecular heterogeneity and microenvironmental characteristics of breast cancer. Furthermore, ST has facilitated the identification of key signaling pathways and immune evasion mechanisms during breast cancer progression and metastasis. These discoveries have provided new molecular targets and biomarkers for personalized treatment and prognostic assessment. Additionally, the integration of ST with other omics technologies, such as single-cell transcriptomics, proteomics, and metabolomics, has advanced multidimensional and multilayered research in breast cancer, shedding new light on the complex regulatory mechanisms of the tumor microenvironment. Looking ahead, with advancements in imaging technologies, artificial intelligence algorithms, and molecular preservation techniques, ST is poised to achieve breakthroughs in resolution, detection depth, and data processing capabilities, paving the way for transformative progress in precision medicine and translational research for breast cancer.

In conclusion, while ST faces significant challenges in terms of technical limitations, data analysis complexity, and sample preparation, its future is bright. Advances in imaging, sequencing, and computational technologies are poised to overcome these hurdles, paving the way for breakthroughs in resolution, detection depth, and data processing. The clinical potential of ST, particularly in personalized medicine and prognostic assessment, is immense, and its integration with other omics technologies will enable a more holistic understanding of biological systems. As ST continues to mature, it promises to revolutionize precision medicine and disease research, ultimately contributing to improved human health and well-being.

## Figures and Tables

**Table 1:** Comparative table of spatial transcriptomics technologies.

Technology Type	Representative Methods	Principle	Resolution	Gene Capacity	Advantages	Limitations	References
In Situ Sequencing (ISS)	FISSEQ, STARMap	Direct in situ sequencing (sequencing-by-ligation/synthesis) of RNA in tissue	single molecule	Targeted (~50–1000 genes)	High resolution, no RNA extraction	Low detection efficiency	[27,32]
In Situ Hybridization (ISH)	MERFISH, SeqFISH	Multiplexed fluorescent probe hybridization + barcode decoding	single cell	High-plex (~10,000 genes)	Ultra-high resolution, multi-target detection	Complex workflow, requires high-end imaging	[34,35,36,37]
NGS-Based	GeoMx DSP	UV-cleavable probes for region-specific mRNA capture	10 μm	Targeted/Whole Transcriptome	FFPE-compatible, flexible region selection	May create bias in selecting regions	[47]
	LCM + RNA-seq	Laser-capture microdissection + RNA-seq	Cellular	Whole Transcriptome	High sensitivity	Low throughput, destructive sampling	[22]
	10× Visium	Spatial barcoded array for poly-A mRNA capture	55 µm (HD: 2 µm)	Whole Transcriptome	Whole transcriptome, commercial maturity	Limited resolution (mixed cells)	[50]
	Slide-seqV2	10 µm barcoded bead array	10 µm	Whole Transcriptome	High resolution	Low mRNA capture efficiency (~30–50% of scRNA-seq)	[52]
	Stereo-seq	Nanoscale barcoded RCA (rolling circle amplification)	0.22 µm	Whole Transcriptome	Ultrahigh resolution, large field-of-view	Massive data, complex analysis	[53]
	DBiT-seq	Orthogonal microfluidic barcode printing (RNA + protein)	10–50 µm	Whole Transcriptome + Protein	Multi-omics integration, minimizes mRNA diffusion	Requires specialized equipment	[56]
	XYZeq, sci-Space	Spatial barcoding of intact cells + scRNA-seq	80–500 µm	Whole Transcriptome	Combines scRNA-seq advantages	Requires specialized device	[57,58]

**Table 2:** Application of ST in tumor heterogeneity of breast cancer.

Study Subject	Category	Technique	Key Genes, Pathways, or Cell Populations	Clinical Significance	References
Different origins	Ductal and lobular epithelial cells	GeoMx DSP and snRNA-seq	• 10 differentially expressed genes. • MAPKK signaling pathway. • Sin3a-histone deacetylase complex.	Highlighting the spatial heterogeneity of the ductal and lobular tissue regions and age-related mechanisms of breast cancer.	[60,61]
	MDLC	GeoMx DSP	• MYC signaling pathway. • ER signaling pathway.	• Emphasizing the molecular heterogeneity between ductal and lobular tumor regions. • Proposing personalized treatment strategies.	[62]
Different molecular subtypes	HR+	10×Visium and GeoMx DSP	• Proliferative cell populations. • Estrogen-responsive cell populations. • Hypoxia-induced compartments. • Inflammation-related compartments. • Ribosome-related genes. • Estrogen signaling pathway.	• Establishing a molecular basis for luminal breast cancer precision therapy. • Proposing age-specific therapeutic strategies for breast cancer.	[63,64]
	HER2+	10×Visium	• Metabolic pathway gradients. • Immunosuppressive signaling. • EMT gene modules.	• Establishing a novel spatial omics framework. • Investigating tumor ecosystem dynamics. • Discovering precision immunotherapy target.	[65]
	TNBC	10×Visium	• Hypoxia-related tumor cells. • Immune-enriched regions.	• Providing new insights into personalized treatment for TNBC. • Targeting hypoxia and the TIME for therapy.	[66]
Special histological subtypes	PTs	scRNA-seq and 10× Visium	COL4A1/2, CSRP1.	Establishing a molecular basis for precise PTs diagnosis and treatment.	[67]
	IMPC	10×Visium	• SREBF1, FASN. • Lipid metabolism. • Glycolytic metabolism.	• Providing new insights into the aggressive behavior of IMPC. • Paving the way for precise breast cancer diagnosis and treatment.	[68]
	BRCA1/2 mutation carriers	GeoMx DSP	• MMP3, MMP8. • Integrin-mediated mechanotransduction mechanisms.	Laying the groundwork for precision therapies in BRCA1/2-related breast cancer.	[69]
Other dimensions	Two key axes: EMT and luminal-basal (lineage) plasticity	scRNA-seq and 10×Visium	• Epithelial characteristic markers. • Mesenchymal characteristic markers.	• Deepening insights into breast cancer heterogeneity and clinical impact. • Uncovering the interplay between EMT and tamoxifen resistance.	[71]
	Lymph node-positive vs. negative breast cancer	GeoMx DSP	• NR4A1, JUN. • Myeloid differentiation. • Mononuclear cell differentiation. • Hematopoietic regulation.	Highlighting the need for personalized therapies in breast cancer with lymph node metastasis.	[72]
	9 major cell types with multiple functional states	10×Visium	• Proliferative core. • EMT-enriched invasive front. • Lipid metabolism, oxidative phosphorylation. • Immunosuppressive niches.	A roadmap to overcome breast cancer heterogeneity through microenvironment-informed precision targeting.	[73]

Abbreviations: MDLC, mixed invasive ductal and lobular carcinoma; HR, hormone receptor; HER2, human epidermal growth factor receptor 2; TNBC, triple-negative breast cancer; PTs, breast phyllodes tumors; IMPC, invasive micropapillary carcinoma; EMT, epithelial-mesenchymal transition.

**Table 4:** Applications of ST in spatial dynamic alterations during the progression and metastasis of breast cancer.

Classification	Study Subject	Technique	Main Findings	References
Spatial Dynamic Alterations during Progression	Normal tissue to tumor tissue	10× Visium	• Increased immune cell infiltration in tumor tissues; spatial co-localization of epithelial and immune cells.	[88]
	DCIS to IDC	Not mentioned	• Increased CNV and activity of tumor-related signaling pathways. • Elevated proportions of Treg and CXCL13+ T cells. • T cell exhaustion.	[89]
	ER+ breast cancer	10× Visium	• Increased proportion of epithelial cells; immune cells enriched around epithelial cells. • Malignant cells interact with immune cells via ligand–receptor pairs.	[90]
Spatial Dynamic Alterations during Metastasis	TME remodeling during tumor metastasis	scRNA-seq and 10× Visium	• EDCs undergo metabolic switching during metastasis. • EDCs interact with immune cells via the MIF pathway.	[91]
	Spatial heterogeneity of lymph nodes in metastatic breast cancer	bulk RNA-seq and 10× Visium	• CD169+ macrophages are suppressed and eliminated in metastatic lymph nodes, impairing anti-tumor immune responses. • Reduction in CD169+ macrophages correlates with breast cancer pathological staging.	[92]

Abbreviations: DCIS, ductal carcinoma in situ; IDC, invasive ductal carcinoma; CNV, copy number variation; TIME, tumor immune microenvironment; TNBC, triple-negative breast cancer; TILs, tumor-infiltrating lymphocytes; TME, tumor microenvironment; ER, estrogen receptor; EDCs, early disseminated tumor cells.

**Table 5:** Applications of ST in breast cancer treatment.

Classification	Study Subject	Technique	Main Findings	Clinical Significance	References
Chemotherapy	Protein expression linked to chemosensitivity and better prognosis differs between stromal and tumor regions	GeoMx DSP	• Stromal region: ERα ↑ 4-1BB ↓ MART1 ↓GITR ↑ • Tumor region: GZMA ↑ STING ↑ fibronectin ↑ CD80 ↓ PD-L1 ↑ FOXP3 ↑ NF1 ↑	Insights for TNBC precision treatment.	[93]
NAT	TME changes before/after NAC	Not mentioned	• In pCR patients: TILs ↑ IFN genes↑ • In pNR patients: angiogenesis/oxidative metabolism↑	Revealing NAC response mechanisms.	[94]
	TME changes before/after NAT (combined radiotherapy and immunotherapy)	scRNA-seq and 10× Visium	• After therapy: CD8+ T cells ↑ macrophages ↑ • B-cell/T-cell regions linked to response	Basis for combining immunotherapy and radiotherapy.	[95]
Immunotherapy	KLF5/COX2/PGE2 axis in the efficacy of anti-PD1 therapy	10× Visium	• KLF5 deletion enhances CD8+ T cell infiltration. • KLF5 inhibits CD8+ T cells via COX2/PGE2.	KLF5/COX2/PGE2 axis as a therapeutic target.	[96]
	TME Characteristics by PD-L1 status in TNBC	10× Visium	• PD-L1+ tumors: M2 macrophages ↑ HLA II genes ↑ • PD-L1- tumors: immune suppression ↑	Guiding immunotherapy optimization.	[97]
	The role of macrophages in immunotherapy	10× Visium	• Tryptophan metabolism drives M1 polarization. • High tryptophan metabolism enhances immune scores.	Tryptophan metabolism as a new immunotherapy target.	[98]
Combination of immunotherapy and other treatment strategies	Combination of PTX and PD-1 blockade	10× Visium	• PTX activates TLR4+ TAMs, boosting CD8+ T cells. • PTX + PD1 blockade synergizes tumor regression.	TLR4+ TAMs mediate PTX-induced antitumor immunity; new TNBC immunotherapy strategies.	[99]
	Eganelisib in combination with atezolizumab and nab-paclitaxel	mIF and GeoMx DSP	• Immune-activating cytokines ↑ immunosuppressive cytokines↓ • Eganelisib downregulates ECM genes in PD-L1- tumors.	PI3K-γ inhibitors enhance antitumor immunity via TAM reprogramming.	[100]
Chemotherapy resistance	PTX resistance and EC spatial heterogeneity	10× Visium	• EC_sub2 drives immunosuppression. • TNFR2 signaling promotes resistance.	ECs critically mediate PTX resistance.; TNFR2 blockade as a new strategy.	[101]
Resistance to HER2-targeted therapies	Spatial heterogeneity in HER2+ breast cancer	GeoMx DSP	• HER2-high/CK5-high cells spatially exclusive • ER- tumors exhibit elevated spatial heterogeneity.	Spatial heterogeneity impacts HER2+ treatment response.	[102]
	IMM2902 overcomes HER2 resistance	10× Visium	• IMM2902 (CD47/HER2 bispecific) increases immune cell infiltration. • Effective in trastuzumab-resistant models	New strategy for HER2+ breast cancer, especially resistant cases.	[103]
Immune therapy resistance	ITH and immunotherapy resistance in TNBC	ChIP-seq, scRNA-seq, and 10× Visium	• ZNF689 deficiency derepresses LINE-1, increasing ITH and resistance. • EFV (LINE-1 inhibitor) reverses PD-1 resistance.	ZNF689/LINE-1 as targets; “heterogeneity-targeting plus immunotherapy” strategy for high-ITH tumors.	[104]

Abbreviations: TME, tumor microenvironment; ER, estrogen receptor; TNBC, triple-negative breast cancer; TILs, tumor-infiltrating lymphocytes; NAT, neoadjuvant therapy; NAC, neoadjuvant chemotherapy; pCR, pathological complete response; pNR, pathological non-response; HLA, human leukocyte antigen; mIF, multiplex immunofluorescence; PTX, paclitaxel; TAMs, tumor-associated macrophages; ECM, extracellular matrix; EC, endothelial cells; HER2+, human epidermal growth factor receptor 2 positive; ITH, intratumor heterogeneity; ChIP, chromatin immunoprecipitation.

## Data Availability

The corresponding authors will provide the datasets created during and/or analyzed during the current investigation upon reasonable request.

## Abbreviations

CAF, cancer-associated fibroblasts; DCIS, ductal carcinoma in situ; DCs, dendritic cells; DSP, digital spatial profiling; ECTs, EcoCellTypes; EDCs, early disseminated tumor cells; ER+, estrogen receptor positive; FFPE, formalin-fixed paraffin-embedded; HER2+, human epidermal growth factor receptor 2 positive; HR+, hormone receptor positive; IDC, invasive ductal carcinoma; IMPC, invasive micropapillary carcinoma; ITH, intratumor heterogeneity; iCAF, inflammatory cancer-associated fibroblasts; MDLC, mixed invasive ductal and lobular carcinoma; myCAF, myofibroblastic cancer-associated fibroblasts; NAC, neoadjuvant chemotherapy; NAT, neoadjuvant therapy; OS, overall survival; PFS, progression-free survival; PR, progesterone receptor; PTs, phyllodes tumors; pCR, pathological complete response; pNR, pathological non-response; scRNA-seq, single-cell RNA sequencing; ST, spatial transcriptomics; TAMs, tumor-associated macrophages; TILs, tumor-infiltrating lymphocytes; TIME, tumor immune microenvironment; TME, tumor microenvironment; and, Tregs, regulatory T cells.

## References

[1] Menon G., Alkabban F.M., Ferguson T. (2025). Breast Cancer.

[2] Dong B., Yin X., Xu H., Zhou K., Li L., Tian B., Cui R. (2022). Application value of modified radical mastectomy in female patients with breast cancer of different molecular types: A prognosis study. Am. J. Transl. Res..

[3] Zhang Y., Li G., Bian W., Bai Y., He S., Liu Y., Liu H., Liu J. (2022). Value of genomics- and radiomics-based machine learning models in the identification of breast cancer molecular subtypes: A systematic review and meta-analysis. Ann. Transl. Med..

[4] Hashmi A.A., Bukhari U., Najam J., Dowlah T., Ali A.H., Diwan M.A., Anjali F.N., Sham S., Zia S., Irfan M. (2023). Luminal B, Human Epidermal Growth Factor Receptor 2 (HER2/neu), and Triple-Negative Breast Cancers Associated With a Better Chemotherapy Response Than Luminal A Breast Cancers in Postneoadjuvant Settings. Cureus.

[5] Ganguly A., Mukherjee S., Chatterjee K., Spada S. (2024). Factors affecting heterogeneity in breast cancer microenvironment: A narrative mini review. Int. Rev. Cell Mol. Biol..

[6] Lüönd F., Tiede S., Christofori G. (2021). Breast cancer as an example of tumour heterogeneity and tumour cell plasticity during malignant progression. Br. J. Cancer.

[7] Thakur S., Haider S., Natrajan R. (2023). Implications of tumour heterogeneity on cancer evolution and therapy resistance: Lessons from breast cancer. J. Pathol..

[8] Rodríguez-Bejarano O.H., Parra-López C., Patarroyo M.A. (2024). A review concerning the breast cancer-related tumour microenvironment. Crit. Rev. Oncol. Hematol..

[9] Benvenuto M., Focaccetti C. (2024). Tumor Microenvironment: Cellular Interaction and Metabolic Adaptations. Int. J. Mol. Sci..

[10] Jin Z., Zhou Q., Cheng J.-N., Jia Q., Zhu B. (2023). Heterogeneity of the tumor immune microenvironment and clinical interventions. Front. Med..

[11] Sheng W., Zhang C., Mohiuddin T.M., Al-Rawe M., Zeppernick F., Falcone F.H., Meinhold-Heerlein I., Hussain A.F. (2023). Multiplex Immunofluorescence: A Powerful Tool in Cancer Immunotherapy. Int. J. Mol. Sci..

[12] Li Y., Jin J., Bai F. (2022). Cancer biology deciphered by single-cell transcriptomic sequencing. Protein Cell.

[13] Hu J., Chen Z., Bao L., Zhou L., Hou Y., Liu L., Xiong M., Zhang Y., Wang B., Tao Z. (2024). Single-cell transcriptome analysis reveals intratumoral heterogeneity in lung adenocarcinoma. Environ. Toxicol..

[14] Kaminska B., Ochocka N., Segit P. (2021). Single-Cell Omics in Dissecting Immune Microenvironment of Malignant Gliomas—Challenges and Perspectives. Cells.

[15] Zhang Y., Gong S., Liu X. (2024). Spatial transcriptomics: A new frontier in accurate localization of breast cancer diagnosis and treatment. Front. Immunol..

[16] Tian L., Chen F., Macosko E.Z. (2023). The expanding vistas of spatial transcriptomics. Nat. Biotechnol..

[17] Wang Y., Liu B., Zhao G., Lee Y., Buzdin A., Mu X., Zhao J., Chen H., Li X. (2023). Spatial transcriptomics: Technologies, applications and experimental considerations. Genomics.

[18] Xue R., Zhang Q., Cao Q., Kong R., Xiang X., Liu H., Feng M., Wang F., Cheng J., Li Z. (2022). Liver tumour immune microenvironment subtypes and neutrophil heterogeneity. Nature.

[19] Xun Z., Ding X., Zhang Y., Zhang B., Lai S., Zou D., Zheng J., Chen G., Su B., Han L. (2023). Reconstruction of the tumor spatial microenvironment along the malignant-boundary-nonmalignant axis. Nat. Commun..

[20] Moffitt J.R., Lundberg E., Heyn H. (2022). The emerging landscape of spatial profiling technologies. Nat. Rev. Genet..

[21] Li Z., Peng G. (2022). Spatial transcriptomics: New dimension of understanding biological complexity. Biophys. Rep..

[22] Moses L., Pachter L. (2022). Museum of spatial transcriptomics. Nat. Methods.

[23] Ke R., Mignardi M., Pacureanu A., Svedlund J., Botling J., Wählby C., Nilsson M. (2013). In situ sequencing for RNA analysis in preserved tissue and cells. Nat. Methods.

[24] Darmanis S., Sloan S.A., Croote D., Mignardi M., Chernikova S., Samghababi P., Zhang Y., Neff N., Kowarsky M., Caneda C. (2017). Single-Cell RNA-Seq Analysis of Infiltrating Neoplastic Cells at the Migrating Front of Human Glioblastoma. Cell Rep..

[25] Carow B., Hauling T., Qian X., Kramnik I., Nilsson M., Rottenberg M.E. (2019). Spatial and temporal localization of immune transcripts defines hallmarks and diversity in the tuberculosis granuloma. Nat. Commun..

[26] Tiklova K., Bjorklund A.K., Lahti L., Fiorenzano A., Nolbrant S., Gilberg L., Volakakis N., Yokota C., Hilscher M., Hauling T. (2019). Single-cell RNA sequencing reveals midbrain dopamine neuron di-versity emerging during mouse brain development. Nat. Commun..

[27] Wang X., Allen W.E., Wright M.A., Sylwestrak E.L., Samusik N., Vesuna S., Evans K., Liu C., Ramakrishnan C., Liu J. (2018). Three-dimensional intact-tissue sequencing of single-cell transcriptional states. Science.

[28] Chen X., Sun Y.-C., Church G.M., Lee J.H., Zador A.M. (2018). Efficient in situ barcode sequencing using padlock probe-based BaristaSeq. Nucleic Acids Res..

[29] Chen X., Sun Y.C., Zhan H., Kebschull J.M., Fischer S., Matho K., Huang Z.J., Gillis J., Zador A.M. (2019). High-Throughput Mapping of Long-Range Neuronal Projection Using In Situ Sequencing. Cell.

[30] Gyllborg D., Langseth C.M., Qian X., Choi E., Salas S.M., Hilscher M.M., Lein E.S., Nilsson M. (2020). Hybridization-based in situ sequencing (HybISS) for spatially re-solved transcriptomics in human and mouse brain tissue. Nucleic Acids Res..

[31] Alon S., Goodwin D.R., Sinha A., Wassie A.T., Chen F., Daugharthy E.R., Bando Y., Kajita A., Xue A.G., Marrett K. (2021). Expansion sequencing: Spatially precise in situ transcriptomics in intact biological systems. Science.

[32] Lee J.H., Daugharthy E.R., Scheiman J., Kalhor R., Yang J.L., Ferrante T.C., Terry R., Jeanty S.S.F., Li C., Amamoto R. (2014). Highly multiplexed subcellular RNA sequencing in situ. Science.

[33] Codeluppi S., Borm L.E., Zeisel A., La Manno G., van Lunteren J.A., Svensson C.I., Linnarsson S. (2018). Spatial organization of the somatosensory cortex revealed by osmFISH. Nat. Methods.

[34] Wang G., Moffitt J.R., Zhuang X. (2018). Multiplexed imaging of high-density libraries of RNAs with MERFISH and expansion microscopy. Sci. Rep..

[35] Xia C., Babcock H.P., Moffitt J.R., Zhuang X. (2019). Multiplexed detection of RNA using MERFISH and branched DNA amplification. Sci. Rep..

[36] Moffitt J.R., Bambah-Mukku D., Eichhorn S.W., Vaughn E., Shekhar K., Perez J.D., Rubinstein N.D., Hao J., Regev A. (2018). Molecular, spatial, and functional single-cell profiling of the hypothalamic preoptic region. Science.

[37] Lohoff T., Ghazanfar S., Missarova A., Koulena N., Pierson N., Griffiths J.A., Bardot E.S., Eng C.-H.L., Tyser R.C.V., Argelaguet R. (2022). Integration of spatial and single-cell transcriptomic data elucidates mouse organogenesis. Nat. Biotechnol..

[38] Takei Y., Yun J., Zheng S., Ollikainen N., Pierson N., White J., Shah S., Thomassie J., Suo S., Eng C.-H.L. (2021). Integrated spatial genomics reveals global architecture of single nuclei. Nature.

[39] Eng C.H., Lawson M., Zhu Q., Dries R., Koulena N., Takei Y., Yun J., Cronin C., Karp C., Yuan G.C. (2019). Transcriptome-scale super-resolved imaging in tissues by RNA seqFISH. Nature.

[40] Asp M., Bergenstråhle J., Lundeberg J. (2020). Spatially Resolved Transcriptomes—Next Generation Tools for Tissue Exploration. BioEssays.

[41] Zahedi R., Ghamsari R., Argha A., Macphillamy C., Beheshti A., Alizadehsani R., Lovell N.H., Lotfollahi M. (2024). Deep learning in spatially resolved transcriptfomics: A comprehensive technical view. Brief Bioinform..

[42] Park J., Choi W., Tiesmeyer S., Long B., Borm L.E., Garren E., Nguyen T.N., Tasic B., Codeluppi S., Graf T. (2021). Cell segmentation-free inference of cell types from in situ transcriptomics data. Nat. Commun..

[43] Littman R., Hemminger Z., Foreman R., Arneson D., Zhang G., Gómez-Pinilla F., Yang X., Wollman R. (2021). Joint cell segmentation and cell type annotation for spatial transcriptomics. Mol. Syst. Biol..

[44] Qian X., Harris K.D., Hauling T., Nicoloutsopoulos D., Muñoz-Manchado A.B., Skene N., Hjerling-Leffler J., Nilsson M. (2020). Probabilistic cell typing enables fine mapping of closely related cell types in situ. Nat. Methods.

[45] Kruse F., Junker J.P., Van Oudenaarden A., Bakkers J. (2016). Tomo-seq: A method to obtain genome-wide expression data with spatial resolution. Methods Cell Biol..

[46] Chen J., Suo S., Tam P.P., Han J.-D.J., Peng G., Jing N. (2017). Spatial transcriptomic analysis of cryosectioned tissue samples with Geo-seq. Nat. Protoc..

[47] Merritt C.R., Ong G.T., Church S.E., Barker K., Danaher P., Geiss G., Hoang M., Jung J., Liang Y., McKay-Fleisch J. (2020). Multiplex digital spatial profiling of proteins and RNA in fixed tissue. Nat. Biotechnol..

[48] Schede H.H., Schneider C.G., Stergiadou J., Borm L.E., Ranjak A., Yamawaki T.M., David F.P., Lönnerberg P., Tosches M.A. (2021). Spatial tissue profiling by imaging-free molecular tomography. Nat. Biotechnol..

[49] Li Y.-H., Cao Y., Liu F., Zhao Q., Adi D., Huo Q., Liu Z., Luo J.-Y., Fang B.-B., Tian T. (2021). Visualization and Analysis of Gene Expression in Stanford Type A Aortic Dissection Tissue Section by Spatial Transcriptomics. Front. Genet..

[50] Polański K., Bartolomé-Casado R., Sarropoulos I., Xu C., England N., Jahnsen F.L., A Teichmann S., Yayon N., Mathelier A. (2024). Bin2cell reconstructs cells from high resolution Visium HD data. Bioinformatics.

[51] Rodriques S.G., Stickels R.R., Goeva A., Martin C.A., Murray E., Vanderburg C.R., Welch J., Chen L.M., Chen F., Macosko E.Z. (2019). Slide-seq: A scalable technology for measuring genome-wide expression at high spatial resolution. Science.

[52] Stickels R.R., Murray E., Kumar P., Li J., Marshall J.L., Di Bella D.J., Arlotta P., Macosko E.Z., Chen F. (2021). Highly sensitive spatial transcriptomics at near-cellular resolution with Slide-seqV2. Nat. Biotechnol..

[53] Chen A., Liao S., Cheng M., Ma K., Wu L., Lai Y., Qiu X., Yang J., Xu J., Hao S. (2022). Spatiotemporal transcriptomic atlas of mouse organogenesis using DNA nano-ball-patterned arrays. Cell.

[54] Kleino I., Frolovaitė P., Suomi T., Elo L.L. (2022). Computational solutions for spatial transcriptomics. Comput. Struct. Biotechnol. J..

[55] Villacampa E.G., Larsson L., Mirzazadeh R., Kvastad L., Andersson A., Mollbrink A., Kokaraki G., Monteil V., Schultz N. (2021). Genome-wide spatial expression profiling in for-malin-fixed tissues. Cell Genom..

[56] Liu Y., Yang M., Deng Y., Su G., Enninful A., Guo C.C., Tebaldi T., Zhang D., Kim D., Bai Z. (2020). High-Spatial-Resolution Multi-Omics Sequencing via Deterministic Barcoding in Tissue. Cell.

[57] Lee Y., Bogdanoff D., Wang Y., Hartoularos G.C., Woo J.M., Mowery C.T., Nisonoff H.M., Lee D.S., Sun Y., Lee J. (2021). XYZeq: Spatially resolved single-cell RNA sequencing reveals expression heterogeneity in the tumor microenvironment. Sci. Adv..

[58] Srivatsan S.R., Regier M.C., Barkan E., Franks J.M., Packer J.S., Grosjean P., Duran M., Saxton S., Ladd J.J., Spielmann M. (2021). Embryo-scale, single-cell spatial transcriptomics. Science.

[59] An J., Lu Y., Chen Y., Chen Y., Zhou Z., Chen J., Peng C., Huang R., Peng F. (2024). Spatial transcriptomics in breast cancer: Providing insight into tumor heterogeneity and promoting individualized therapy. Front. Immunol..

[60] Bhat-Nakshatri P., Gao H., Khatpe A.S., Adebayo A.K., McGuire P.C., Erdogan C., Chen D., Jiang G., New F., German R. (2024). Single-nucleus chromatin accessibility and transcriptomic map of breast tissues of women of diverse genetic ancestry. Nat. Med..

[61] Liu H.-L., Lu X.-M., Wang H.-Y., Hu K.-B., Wu Q.-Y., Liao P., Li S., Long Z.-Y., Wang Y.-T. (2023). The role of RNA splicing factor PTBP1 in neuronal development. Biochim. Biophys. Acta-Mol. Cell Res..

[62] Shah O.S., Nasrazadani A., Foldi J., Atkinson J.M., Kleer C.G., McAuliffe P.F., Johnston T.J., Stallaert W., da Silva E.M., Selenica P. (2024). Spatial molecular profiling of mixed invasive ductal and lobular breast cancers reveals heterogeneity in intrinsic molecular subtypes, oncogenic signatures, and mutations. Proc. Natl. Acad. Sci. USA.

[63] Yoshitake R., Mori H., Ha D., Wu X., Wang J., Wang X., Saeki K., Chang G., Shim H.J., Chan Y. (2024). Molecular features of luminal breast cancer defined through spatial and single-cell transcriptomics. Clin. Transl. Med..

[64] Chu J., Do S.-I., Kim H.-S. (2024). Age-related Differences in Spatially Resolved Transcriptomic Profiles of Patients with Hormone Receptor-positive Breast Carcinoma. Anticancer. Res..

[65] Andersson A., Larsson L., Stenbeck L., Salmén F., Ehinger A., Wu S.Z., Al-Eryani G., Roden D., Swarbrick A., Borg Å. (2021). Spatial deconvolution of HER2-positive breast cancer delineates tumor-associated cell type interactions. Nat. Commun..

[66] Bassiouni R., Idowu M.O., Gibbs L.D., Robila V., Grizzard P.J., Webb M.G., Song J., Noriega A., Craig D.W., Carpten J.D. (2023). Spatial Transcriptomic Analysis of a Diverse Patient Cohort Reveals a Conserved Architecture in Triple-Negative Breast Cancer. Cancer Res..

[67] Li X., Yu X., Bi J., Jiang X., Zhang L., Li Z., Shao M. (2024). Integrating single-cell and spatial transcriptomes reveals COL4A1/2 facilitates the spatial organisation of stromal cells differentiation in breast phyllodes tumours. Clin. Transl. Med..

[68] Lv J., Shi Q., Han Y., Li W., Liu H., Zhang J., Niu C., Gao G., Fu Y., Zhi R. (2021). Spatial transcriptomics reveals gene expression characteristics in invasive micropapillary carcinoma of the breast. Cell Death Dis..

[69] Caputo A., Vipparthi K., Bazeley P., Downs-Kelly E., McIntire P., Duckworth L.A., Ni Y., Hu B., Keri R.A., Karaayvaz M. (2024). Spatial Transcriptomics Suggests That Alterations Occur in the Pre-neoplastic Breast Microenvironment of BRCA1/2 Mutation Carriers. Mol. Cancer Res..

[70] van Boxtel C., van Heerden J.H., Nordholt N., Schmidt P., Bruggeman F.J. (2017). Taking chances and making mistakes: Non-genetic pheno-typic heterogeneity and its consequences for surviving in dynamic environments. J. R. Soc. Interface.

[71] Sahoo S., Ramu S., Nair M.G., Pillai M., Juan B.P.S., Milioli H.Z., Mandal S., Naidu C.M., Mavatkar A.D., Subramaniam H. (2024). Increased prevalence of hybrid epithelial/mesenchymal state and enhanced phenotypic heterogeneity in basal breast cancer. iScience.

[72] Chung Y., Chu J., Do S.-I., Kim H.-S. (2024). Spatial Transcriptomic Profiling Reveals Gene Expression Characteristics in Lymph Node-positive Breast Carcinoma. Anticancer. Res..

[73] Wu S.Z., Al-Eryani G., Roden D.L., Junankar S., Harvey K., Andersson A., Thennavan A., Wang C., Torpy J.R., Bartonicek N. (2021). A single-cell and spatially resolved atlas of human breast cancers. Nat. Genet..

[74] Jin M.-Z., Jin W.-L. (2020). The updated landscape of tumor microenvironment and drug repurposing. Signal Transduct. Target. Ther..

[75] Han M., Li S., Fan H., An J., Peng C., Peng F. (2024). Regulated cell death in glioma: Promising targets for natural small-molecule compounds. Front. Oncol..

[76] Romanens L., Chaskar P., Marcone R., Ryser S., Tille J., Genolet R., Heimgartner-Hu K., Heimgartner K., Moore J.S., Liaudet N. (2023). Clonal expansion of intra-epithelial T cells in breast cancer revealed by spatial transcriptomics. Int. J. Cancer.

[77] Wu B., Li L., Li L., Chen Y., Guan Y., Zhao J. (2024). Integration of Bioinformatics and Machine Learning to Identify CD8+ T Cell-Related Prognostic Signature to Predict Clinical Outcomes and Treatment Response in Breast Cancer Patients. Genes.

[78] Yu X., Li W., Sun S., Li J. (2024). DDIT3 is associated with breast cancer prognosis and immune microenvironment: An inte-grative bioinformatic and immunohistochemical analysis. J. Cancer.

[79] Tzeng Y.D., Chu P.Y., Yong S.B., Hsu T.S., Tseng L.M., Hou M.F., Sheu J.J., Hsiao J.H., Li C.J. (2024). Multi-omic profiling of breast tumor microenvironment uncovers a role of mito-chondrial calcium gatekeepers. J. Cancer.

[80] Liu G., Wang L., Ji L., He D., Zeng L., Zhuo G., Zhang Q., Wang D., Pan Y. (2023). Identifying prognostic markers in spatially heterogeneous breast cancer microenvironment. J. Transl. Med..

[81] Croizer H., Mhaidly R., Kieffer Y., Gentric G., Djerroudi L., Leclere R., Pelon F., Robley C., Bohec M., Meng A. (2024). Deciphering the spatial landscape and plasticity of immunosuppressive fibroblasts in breast cancer. Nat. Commun..

[82] Honda C.K., Kurozumi S., Fujii T., Pourquier D., Khellaf L., Boissiere F., Horiguchi J., Oyama T., Shirabe K., Colinge J. (2024). Cancer-associated fibroblast spatial heterogeneity and *EMILIN1* expression in the tumor microenvironment modulate TGF-β activity and CD8^+^ T-cell infiltration in breast cancer. Theranostics.

[83] Ma C., Yang C., Peng A., Sun T., Ji X., Mi J., Wei L., Shen S., Feng Q. (2023). Pan-cancer spatially resolved single-cell analysis reveals the crosstalk between can-cer-associated fibroblasts and tumor microenvironment. Mol. Cancer.

[84] Chen M., Liu H., Hong B., Xiao Y., Qian Y. (2024). MIF as a potential diagnostic and prognostic biomarker for triple-negative breast cancer that correlates with the polarization of M2 macrophages. FASEB J..

[85] Zhu Y., Banerjee A., Xie P., Ivanov A.A., Uddin A., Jiao Q., Chi J.J., Zeng L., Lee J.Y., Xue Y. (2024). Pharmacological suppression of the OTUD4/CD73 proteolytic axis revives anti-tumor immunity against immune-suppressive breast cancers. J. Clin. Investig..

[86] Janesick A., Shelansky R., Gottscho A.D., Wagner F., Williams S.R., Rouault M., Beliakoff G., Morrison C.A., Oliveira M.F., Sicherman J.T. (2023). High resolution mapping of the tumor microenvironment using integrated single-cell, spatial and in situ analysis. Nat. Commun..

[87] Tzeng Y.D., Hsiao J.H., Chu P.Y., Tseng L.M., Hou M.F., Tsang Y.L., Shao A.N., Sheu J.J., Li C.J. (2023). The role of LSM1 in breast cancer: Shaping metabolism and tumor-associated macrophage infiltration. Pharmacol. Res..

[88] Mao X., Zhou D., Lin K., Zhang B., Gao J., Ling F., Zhu L., Yu S., Chen P., Zhang C. (2023). Single-cell and spatial transcriptome analyses revealed cell heterogeneity and immune environment alternations in metastatic axillary lymph nodes in breast cancer. Cancer Immunol. Immunother..

[89] Cai F., Li Y., Liu H., Luo J. (2024). Single-cell and Spatial Transcriptomic Analyses Implicate Formation of the Immunosuppressive Microenvironment during Breast Tumor Progression. J. Immunol..

[90] Xu K., Yu D., Zhang S., Chen L., Liu Z., Xie L. (2024). Deciphering the Immune Microenvironment at the Forefront of Tumor Aggressiveness by Constructing a Regulatory Network with Single-Cell and Spatial Transcriptomic Data. Genes.

[91] Liu Y., Ge J., Chen Y., Liu T., Chen L., Liu C., Ma D., Cai Y., Xu Y., Shao Z. (2023). Combined Single-Cell and Spatial Transcriptomics Reveal the Metabolic Evolvement of Breast Cancer during Early Dissemination. Adv. Sci..

[92] Maeshima Y., Kataoka T.R., Vandenbon A., Hirata M., Takeuchi Y., Suzuki Y., Fukui Y., Kawashima M., Takada M., Ibi Y. (2024). Intra-patient spatial comparison of non-metastatic and met-astatic lymph nodes reveals the reduction of CD169(+) macrophages by metastatic breast cancers. EBioMedicine.

[93] Kulasinghe A., Monkman J., Shah E.T., Matigian N., Adams M.N., O’Byrne K. (2022). Spatial Profiling Identifies Prognostic Features of Response to Adjuvant Therapy in Triple Negative Breast Cancer (TNBC). Front. Oncol..

[94] Donati B., Reggiani F., Torricelli F., Santandrea G., Rossi T., Bisagni A., Gasparini E., Neri A., Cortesi L., Ferrari G. (2024). Spatial Distribution of Immune Cells Drives Resistance to Neoadjuvant Chemotherapy in Triple-Negative Breast Cancer. Cancer Immunol. Res..

[95] Shiao S.L., Gouin K.H., Ing N., Ho A., Basho R., Shah A., Mebane R.H., Zitser D., Martinez A., Mevises N.Y. (2024). Single-cell and spatial profiling identify three response trajectories to pem-brolizumab and radiation therapy in triple negative breast cancer. Cancer Cell.

[96] Wu Q., Liu Z., Gao Z., Luo Y., Li F., Yang C.Y., Wang T., Meng X., Chen H., Li J. (2023). KLF5 inhibition potentiates anti-PD1 efficacy by enhancing CD8^+^ T-cell-dependent antitumor immunity. Theranostics.

[97] Tashireva L.A., Kalinchuk A.Y., Gerashchenko T.S., Menyailo M., Khozyainova A., Denisov E.V., Perelmuter V.M. (2023). Spatial Profile of Tumor Microenvironment in PD-L1-Negative and PD-L1-Positive Triple-Negative Breast Cancer. Int. J. Mol. Sci..

[98] Xue L., Wang C., Qian Y., Zhu W., Liu L., Yang X., Zhang S., Luo D. (2023). Tryptophan metabolism regulates inflammatory macrophage polarization as a predictive factor for breast cancer immunotherapy. Int. Immunopharmacol..

[99] Choi Y., A Kim S., Jung H., Kim E., Kim Y.K., Kim S., Kim J., Lee Y., Jo M.K., Woo J. (2024). Novel insights into paclitaxel’s role on tumor-associated macrophages in enhancing PD-1 blockade in breast cancer treatment. J. Immunother. Cancer.

[100] O’Connell B.C., Hubbard C., Zizlsperger N., Fitzgerald D., Kutok J.L., Varner J., Ilaria R., Cobleigh A.M., Juric D., Tkaczuk K.H.R. (2024). Eganelisib combined with immune checkpoint inhibitor therapy and chemotherapy in frontline metastatic triple-negative breast cancer triggers macrophage reprogramming, immune activation and extracellular matrix reorganization in the tumor microenvironment. J. Immunother. Cancer.

[101] Hu Y., Lou X., Zhang K., Pan L., Bai Y., Wang L., Wang M., Yan Y., Wan J., Yao X. (2024). Tumor necrosis factor receptor 2 promotes endothelial cell-mediated suppression of CD8+ T cells through tuning glycolysis in chemoresistance of breast cancer. J. Transl. Med..

[102] Li Z., Metzger Filho O., Viale G., dell’Orto P., Russo L., Goyette M.A., Kamat A., Yardley D.A., Abramson V.G., Arteaga C.L. (2024). HER2 heterogeneity and treatment response-associated profiles in HER2-positive breast cancer in the NCT02326974 clinical trial. J. Clin. Investig..

[103] Zhang B., Shi J., Shi X., Xu X., Gao L., Li S., Liu M., Gao M., Jin S., Zhou J. (2024). Development and evaluation of a human CD47/HER2 bispecific antibody for Trastuzumab-resistant breast cancer immunotherapy. Drug Resist. Update.

[104] Ge L.-P., Jin X., Ma D., Wang Z.-Y., Liu C.-L., Zhou C.-Z., Zhao S., Yu T.-J., Liu X.-Y., Di G.-H. (2024). ZNF689 deficiency promotes intratumor heterogeneity and immunotherapy resistance in triple-negative breast cancer. Cell Res..

[105] Liao J., Lu X., Shao X., Zhu L., Fan X. (2021). Uncovering an Organ’s Molecular Architecture at Single-Cell Resolution by Spatially Resolved Transcriptomics. Trends Biotechnol..

[106] Dries R., Chen J., del Rossi N., Khan M.M., Sistig A., Yuan G.-C. (2021). Advances in spatial transcriptomic data analysis. Genome Res..

[107] Fu X., Sun L., Dong R., Chen J.Y., Silakit R., Condon L.F., Lin Y., Lin S., Palmiter R.D., Gu L. (2022). Polony gels enable amplifiable DNA stamping and spatial transcriptomics of chronic pain. Cell.

[108] Vickovic S., Eraslan G., Salmén F., Klughammer J., Stenbeck L., Schapiro D., Äijö T., Bonneau R., Bergenstråhle L., Navarro J.F. (2019). High-definition spatial transcriptomics for in situ tissue profiling. Nat. Methods.

[109] He S., Jin Y., Nazaret A., Shi L., Chen X., Rampersaud S., Dhillon B.S., Valdez I., Friend L.E., Fan J.L. (2024). Starfysh integrates spatial transcriptomic and histologic data to reveal heterogeneous tumor–immune hubs. Nat. Biotechnol..

[110] Xu C., Jin X., Wei S., Wang P., Luo M., Xu Z., Yang W., Cai Y., Xiao L., Lin X. (2022). DeepST: Identifying spatial domains in spatial transcriptomics by deep learning. Nucleic Acids Res..

[111] Tanevski J., Flores R.O.R., Gabor A., Schapiro D., Saez-Rodriguez J. (2022). Explainable multiview framework for dissecting spatial relationships from highly multiplexed data. Genome Biol..

[112] Liu N., Bhuva D.D., Mohamed A., Bokelund M., Kulasinghe A., Tan C.W., Davis M.J. (2024). standR: Spatial transcriptomic analysis for GeoMx DSP data. Nucleic Acids Res..

[113] Zappia L., Phipson B., Oshlack A., Schneidman D. (2018). Exploring the single-cell RNA-seq analysis landscape with the scRNA-tools database. PLoS Comput. Biol..

[114] Fang S., Chen B., Zhang Y., Sun H., Liu L., Liu S., Li Y., Xu X. (2023). Computational Approaches and Challenges in Spatial Transcriptomics. Genom. Proteom. Bioinform..

[115] Satija R., A Farrell J., Gennert D., Schier A.F., Regev A. (2015). Spatial reconstruction of single-cell gene expression data. Nat. Biotechnol..

[116] Butler A., Hoffman P., Smibert P., Papalexi E., Satija R. (2018). Integrating single-cell transcriptomic data across different conditions, technologies, and species. Nat. Biotechnol..

[117] Hao Y., Stuart T., Kowalski M.H., Choudhary S., Hoffman P., Hartman A., Srivastava A., Molla G., Madad S. (2024). Dictionary learning for integrative, multimodal and scalable single-cell analysis. Nat. Biotechnol..

[118] Saldanha O.L., Quirke P., West N.P., James J.A., Loughrey M.B., Grabsch H.I., Salto-Tellez M., Alwers E., Cifci D., Ghaffari Laleh N. (2022). Swarm learning for decentralized artificial intelligence in cancer histopathology. Nat. Med..

[119] Shamai G., Livne A., Polónia A., Sabo E., Cretu A., Bar-Sela G., Kimmel R. (2022). Deep learning-based image analysis predicts PD-L1 status from H&E-stained histopathology images in breast cancer. Nat. Commun..

[120] Zubair A., Chapple R.H., Natarajan S., Wright W.C., Pan M., Lee H.-M., Tillman H., Easton J., Geeleher P. (2022). Cell type identification in spatial transcriptomics data can be improved by leveraging cell-type-informative paired tissue images using a Bayesian probabilistic model. Nucleic Acids Res..

[121] Lebrigand K., Bergenstråhle J., Thrane K., Mollbrink A., Meletis K., Barbry P., Waldmann R., Lundeberg J. (2023). The spatial landscape of gene expression isoforms in tissue sections. Nucleic Acids Res..

[122] Khan S., Kim J.J. (2024). Spatial transcriptomics data and analytical methods: An updated perspective. Drug Discov. Today.

[123] Dannhorn A., Kazanc E., Flint L., Guo F., Carter A., Hall A.R., Jones S.A., Poulogiannis G., Barry S.T., Sansom O.J. (2024). Morphological and molecular preservation through universal preparation of fresh-frozen tissue samples for multimodal imaging workflows. Nat. Protoc..

